# Eccentricity rhythms in the Oligocene-Miocene carbon cycle regulated by weathering and carbonate burial

**DOI:** 10.1126/sciadv.adx6682

**Published:** 2026-01-30

**Authors:** Fenghao Liu, Enqing Huang, Jinlong Du, Wentao Ma, Zhonghui Liu, Lucas J. Lourens, Jun Tian

**Affiliations:** ^1^ State Key Laboratory of Marine Geology, Tongji University, Shanghai, China.; ^2^ State Key Laboratory of Satellite Ocean Environment Dynamics, Second Institute of Oceanography, Ministry of Natural Resources, Hangzhou, China.; ^3^ Department of Earth Sciences, The University of Hong Kong, Hong Kong, China.; ^4^ Department of Earth Sciences, Faculty of Geosciences, Utrecht University, Utrecht, Netherlands.

## Abstract

During the Cenozoic unipolar ice ages, benthic foraminiferal oxygen and carbon isotopes (proxies for bottom-water temperature and ice volume and for the carbon cycle, respectively) exhibited in-phase changes on eccentricity timescales. However, the mechanisms underlying this synchronized relationship remain unclear. Here, we present a high-resolution reconstruction of Miocene benthic foraminiferal boron-to-calcium ratios, revealing that eccentricity-paced fluctuations in deep-sea carbonate ion saturation covaried with oxygen and carbon isotopes, as well as with pelagic carbonate deposition. Integrating model results, we propose that orbital configurations and elevated temperatures during eccentricity maxima intensified monsoon rainfall and chemical weathering, enhancing the transport of dissolved inorganic carbon and alkalinity from land to sea. These processes further redistributed massive carbonate burial from deep-ocean basins to continental shelves, lowering carbonate ion concentration and the carbon isotopic composition of seawater. Our findings underscore the crucial role of the low-latitude hydrological cycle in regulating carbon-cycle dynamics under warm climatic conditions.

## Teaser

Continental weathering and shelf-basin carbonate partitioning regulate Oligocene-Miocene carbon cycling on orbital timescales.

## INTRODUCTION

**Fig. 1.**
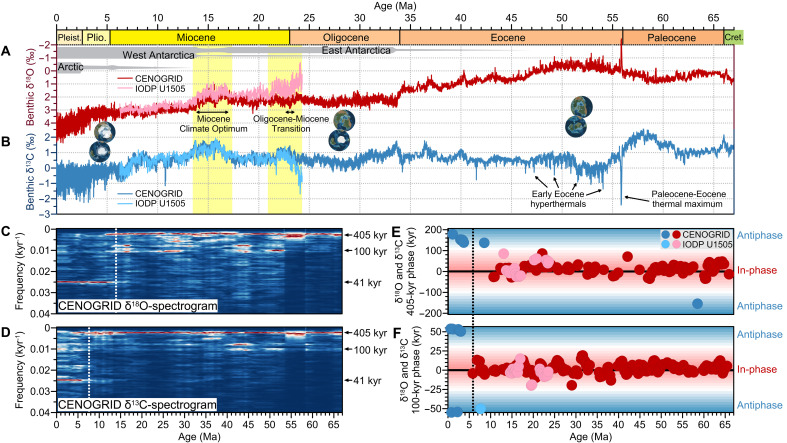
Evolution of astronomical periods in Cenozoic isotope records. (**A** and **B**) Benthic foraminiferal δ^18^O and δ^13^C datasets, with rough estimates of polar ice sheet extent indicated by gray horizontal bars. CENOGRID refers to the Cenozoic Global Reference Isotope Dataset (*2*). (**C** and **D**) Evolutionary multitaper spectrogram was computed with a 5-Myr window on the detrended CENOGRID. From 66 to ~13.9 Ma, cyclic fluctuations in δ^18^O and δ^13^C were dominated by the 405- and 100-kyr eccentricity cycles, whereas the influence of obliquity gradually increased and eventually dominated the variations in both δ^18^O and δ^13^C after ~7.7 Ma. (**E** and **F**) Evolutionary phase relationship between δ^18^O and δ^13^C on eccentricity timescales was in-phase between 66 and ~6 Ma and antiphase after ~6 Ma. Only results with coherence >0.6 are shown. Yellow shading in (A) and (B) highlights the time windows selected for this study.

Global climate change throughout the Cenozoic, the past 66 million years (Myr), has responded to quasi-periodic astronomical forcing (specifically precession, obliquity, and eccentricity) on timescales of 10^4^ to 10^5^ years (*1*). Orbital-scale variations in climate were well documented by stable isotope data from benthic foraminifera (Fig. 1, A to D) (*2*, *3*). Benthic oxygen isotopes (δ^18^O) indicate shifts in deep-sea temperature and ice volume, while carbon isotopes (δ^13^C) track carbon exchange between the ocean and other reservoirs (*3*, *4*). Among these orbital cycles, the imprint of eccentricity, particularly the 405–thousand year (kyr) cycle, was especially prominent in Cenozoic δ^18^O and δ^13^C records (Fig. 1, C and D) (*2*, *5*) despite its relatively minor effect on insolation compared to precession and obliquity (*6*).

During Earth’s ice-free state, from the Cretaceous-Paleogene boundary to the Eocene [66 to 34 million years ago (Ma)], and its unipolar (Antarctic) glaciated state, from the Oligocene to the Late Miocene (34 to ~6 Ma), variations in benthic δ^18^O and δ^13^C exhibited in-phase behavior at 405- and 100-kyr eccentricity cycles (Fig. 1, E and F) (*7*–*10*). In contrast, an antiphase relationship between δ^18^O and δ^13^C emerged during Earth’s transition to a bipolar ice sheet state in the Pliocene and Pleistocene (after ~6 Ma) (Fig. 1, E and F), when the growth and decay of the Northern Hemisphere ice sheets may have governed the net carbon transfer between the terrestrial biosphere and the ocean (*7*, *10*, *11*). This phase reversal, occurring at ~6 Ma, signifies a substantial transformation in the relationship between the temperature-cryosphere system and the carbon cycle (*7*, *10*).

Today, amid accelerating global warming and the ongoing retreat of Northern Hemisphere ice sheets, Earth appears to be transitioning from a bipolar-glacial to a unipolar-glacial regime. Understanding the persistent in-phase δ^18^O-δ^13^C interaction throughout much of the Cenozoic is increasingly critical, yet its drivers are enigmatic. A growing consensus attributes this in-phase pattern to eccentricity-forced modulation of the low-latitude hydrological cycle, which regulated carbon-cycle dynamics (*7*, *9*, *10*, *12*–*15*). Typically, eccentricity maxima corresponded to warm conditions that raised seawater temperatures and reduced the Antarctic cryosphere, resulting in lower deep-sea δ^18^O. Meanwhile, the intensification of monsoon rainfall and chemical weathering in tropical to subtropical regions drove negative excursions in seawater δ^13^C. However, the precise mechanisms linking monsoon intensity to oceanic carbon cycling remain unresolved.

One hypothesis posits that strong monsoons facilitated the transfer of isotopically light carbon (^12^C) from the continental biosphere to the ocean (*7*, *10*). Another suggests that monsoon-driven nutrient inputs may have stimulated the productivity of marine eukaryotic algae, thereby reducing the reservoir of dissolved organic carbon and releasing more ^12^C into the inorganic carbon pool (*10*, *13*, *16*). Testing both ideas remains challenging because of limitations in quantifying terrigenous organic carbon contributions and the absence of proxies for dissolved organic carbon. A third hypothesis proposes that strengthened monsoon rainfall delivered more dissolved inorganic carbon (DIC) and alkalinity (ALK) from land to sea. These influxes promoted considerable accumulation of carbonate on drowned continental shelves and sequestration of isotopically heavy carbon (^13^C) in shallow-water sediments, thus lowering deep-sea δ^13^C (*9*, *12*, *13*). This hypothesis is feasible for investigation, as past changes in the marine carbonate system can be reconstructed.

In this study, we present high-resolution benthic foraminiferal δ^18^O and δ^13^C records spanning the late Oligocene to the Late Miocene (Fig. 1, A and B, light red and blue curves), incorporating new (24.3 to 20.9 Ma) and recently published data (20.9 to 6.3 Ma) (*9*), from the International Ocean Discovery Program (IODP) Site U1505 (18°55.0570′N, 115°51.5370′E; water depth of 2916.6 m) located in the South China Sea (fig. S1 and table S1) (*17*). The ~18-Myr-long isotope record is astronomically age-calibrated [see (*9*), Materials and Methods, and fig. S2]. From the same site, we reconstruct the evolution of deep-water carbonate ion saturation (∆[CO_3_^2−^]) and phosphate concentration ([PO_4_^3−^]) over the intervals 24.3 to 20.9 Ma and 17.4 to 13.5 Ma using benthic foraminiferal boron-to-calcium ratios (B/Ca) and cadmium-to-calcium ratios (Cd/Ca), respectively. Before the Late Miocene, the South China Sea was fully connected to the western Pacific Ocean (fig. S1), accordingly, seawater chemistry at Site U1505 reflected Pacific intermediate/deep water masses (*18*–*20*). In addition, in combination with previously published calcium carbonate (CaCO_3_) data from the Pacific and Atlantic Oceans (table S1) (*5*, *21*–*27*) and our modeling results, we demonstrate that, at least in a unipolar-glaciated world, chemical weathering and shifts in carbonate deposition between continental margins and pelagic environments jointly explain the synchronized δ^18^O-δ^13^C interaction on eccentricity timescales.

## RESULTS

### Orbital imprints in benthic δ^18^O and δ^13^C

**Fig. 2.**
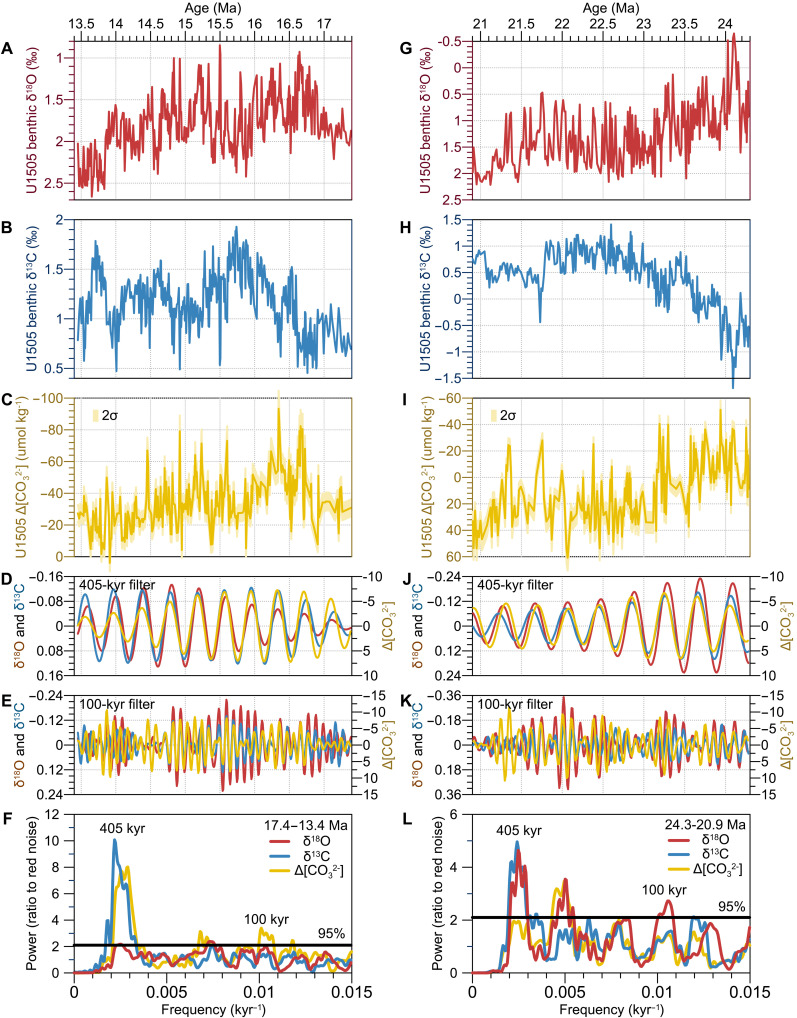
Temporal variations of proxies at Site U1505. (**A** and **G**) Benthic foraminiferal δ^18^O, (**B** and **H**) δ^13^C, and (**C** and **I**) Δ[CO_3_^2−^] records. (**D**, **E**, **J**, and **K**) Bandpass filters of δ^18^O, δ^13^C, and Δ[CO_3_^2−^] in the 405- and 100-kyr bands. The filtering results reveal variations in Δ[CO_3_^2−^] that are in-phase with δ^18^O and δ^13^C within the eccentricity frequency bands. (**F** and **L**) Spectral power of δ^18^O, δ^13^C, and Δ[CO_3_^2−^].

Benthic foraminiferal δ^18^O and δ^13^C records from Site U1505 generally align with the global dataset in both amplitude and absolute values, except during the interval from 24.3 to 20.9 Ma (Figs. 1, A and B, and 2, A, B, G, and H) (*2*, *9*), when the site was situated at a shallower water depth than today due to ongoing subsidence in the South China Sea basin at the time (*17*, *28*). The influence of intermediate water masses during the late Oligocene–Early Miocene likely contributed to relatively low δ^18^O and δ^13^C values (Figs. 1, A and B, and 2, G and H). For the purposes of this study, we set aside this long-term trend to focus on the orbital-scale variability captured by the proxies. Both benthic δ^18^O and δ^13^C display pronounced oscillations on 405- and 100-kyr cycles over 24.3 to 20.9 Ma and 17.4 to 13.5 Ma, with average amplitudes of ~1.2 to 1.5‰ and ~0.8 to 1.4‰ for δ^18^O and ~0.8 to 1.2‰ and ~0.8 to 1.0‰ for δ^13^C, respectively (Fig. 2, D to F and J to L, and fig. S3).

### Eccentricity pacing of deep-ocean Δ[CO_3_^2−^] and [PO_4_^3−^]

The B/Ca and Cd/Ca ratios of the benthic foraminifera *Cibicidoides mundulus* are indicative of deep-water Δ[CO_3_^2−^] (*29*) and [PO_4_^3−^] (*30*), respectively. From 24.3 to 20.9 Ma, Δ[CO_3_^2−^] at Site U1505 oscillates between −51 ± 10 and 61 ± 7 μmol kg^−1^ (uncertainties throughout are reported as two SDs, 2σ), with a mean value of 11 ± 6 μmol kg^−1^ (Fig. 2I). Notably, Δ[CO_3_^2−^] displays an overall increasing trend from −38 ± 6 to 34 ± 8 μmol kg^−1^ between 24.3 and 23.0 Ma (Fig. 2I). This trend, coupled with progressively positive shifts in both δ^18^O and δ^13^C values (Figs. 1, A and B, and 2, G and H), indicates a declining influence of warm, corrosive intermediate waters as the site subsided to greater depths. In the interval 17.4 to 13.5 Ma, Δ[CO_3_^2−^] fluctuates between −93 ± 11 and 4 ± 5 μmol kg^−1^, with an average value of −34 ± 6 μmol kg^−1^(Fig. 2C). At the onset of the Miocene Climate Optimum (~16.9 to 16.6 Ma), which coincides with a ~ 1.0‰ decline in δ^18^O (Figs. 1A and 2A), Δ[CO_3_^2−^] drops rapidly from −8 ± 5 to −82 ± 10 μmol kg^−1^ (Fig. 2C), marking a transition to a super-warm climate regime under high CO_2_ input (*31*–*33*). This decline is followed by a stepwise rise to −27 ± 5 μmol kg^−1^ in Δ[CO_3_^2−^] until ~13.5 Ma (Fig. 2C). In comparison, [PO_4_^3−^] at Site U1505 varies between 1.6 ± 0.13 and 3.5 ± 0.03 μmol kg^−1^, averaging 2.2 ± 0.07 μmol kg^−1^ over the two periods, with no discernible long-term trend (fig. S4, D and J).

Time series analyses reveal that both Δ[CO_3_^2−^] and [PO_4_^3−^] exhibit prominent 405- and 100-kyr cycles throughout the interval 17.4 to 13.5 Ma, but these signals are comparatively weaker between 24.3 and 20.9 Ma (Fig. 2, D to F and J to L, and fig. S3). The weaker eccentricity pacing during the late Oligocene–Early Miocene may also reflect the influence of intermediate waters associated with subsidence of the South China Sea basin. Nonetheless, filtered data suggest persistent eccentricity-band oscillations (Fig. 2, J and K). Across both intervals, the amplitude of these cycles ranges from 30 to 60 μmol kg^−1^ for Δ[CO_3_^2−^] and 0.4 to 0.8 μmol kg^−1^ for [PO_4_^3−^] (fig. S3). Furthermore, Δ[CO_3_^2−^] aligns in phase with δ^18^O and δ^13^C while showing an antiphase relationship with [PO_4_^3−^] on eccentricity timescales (Fig. 2, D, E, J, and K, and fig. S4, E, F, K, and L).

### Global pelagic %CaCO_3_ burial

**Fig. 3.**
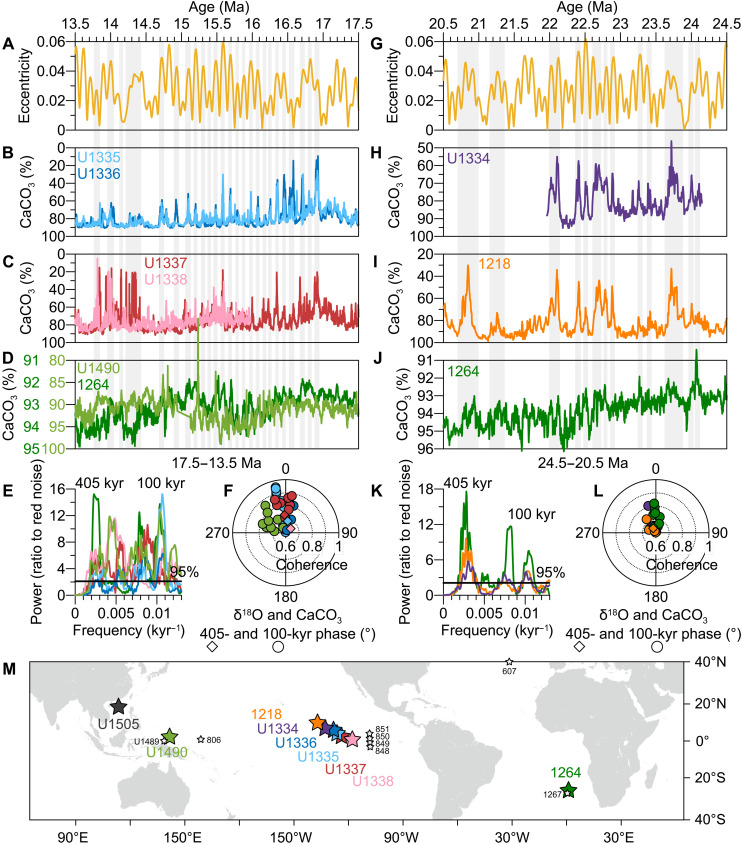
Eccentricity-paced %CaCO_3_ cycles in the deep ocean. (**A** and **G**) Eccentricity (*6*). (**B** to **D** and **H** to **J**) %CaCO_3_ at Pacific Sites 1218, U1334, U1335, U1336, U1337, U1338, U1490, and Atlantic Site 1264 (*5*, *21*–*27*, *34*–*39*). (**E** and **K**) Spectral power of the %CaCO_3_ records. (**F** and **L**) Cross-spectral coherence and phase angles between parallel δ^18^O and %CaCO_3_ records at the 405- and 100-kyr cycles, showing an in-phase relationship. (**M**) Locations of the sites discussed in this study.

We compile seven high-resolution, astronomically tuned records of deep-sea %CaCO_3_ from Ocean Drilling Program (ODP)/IODP sites located in the tropical Pacific and the southeastern Atlantic (*5*, *21*–*27*), spanning the time intervals examined in this study (Fig. 3 and table S1) (*5*, *21*–*24*, *34*–*39*). All %CaCO_3_ data depict pronounced variations in the 405- and 100-kyr eccentricity bands (Fig. 3, E and K) and display in-phase interaction with their respective δ^18^O and δ^13^C series (Fig. 3, F and L, and fig. S5) (*5*, *23*, *24*, *34*–*36*, *38*–*41*). At the eastern Pacific sites (Fig. 3, B, C, H, and I), intense %CaCO_3_ dissolution occurs at the 100-kyr eccentricity maxima, with peak losses ranging from ~10 to 75%. At western Pacific Site U1490 (Fig. 3D) and Atlantic Site 1264 (Fig. 3, D and J), %CaCO_3_ variability is more subdued, with eccentricity-scale amplitudes of ~5 to 10% and ~1 to 4%, respectively.

### Carbon cycle modeling

We use a seven-box biogeochemical model (*9*, *12*) to simulate marine carbon cycling on eccentricity timescales. The model consists of one atmosphere box and six ocean boxes (fig. S6A). Atmospheric CO_2_ is supplied by volcanic and kerogen degassing (fig. S6B). The ocean receives riverine inputs of DIC, ALK, and PO_4_^3−^ derived from chemical weathering, whose fluxes are responsive to atmospheric CO_2_ (fig. S6B). These inputs regulate surface primary productivity, organic carbon burial, and CaCO_3_ deposition and dissolution (fig. S6B). The model is run for 2 Myr from its initial state until equilibrium is achieved, after which it is forced by orbital variations expressed as ETP, the sum of normalized eccentricity, obliquity, and precession (E + T − P) (*6*), and continued for an additional 4 Myr. We conduct one integrated experiment to represent the fully coupled system, along with four sensitivity experiments aimed at isolating the respective roles of riverine DIC and ALK inputs, PO_4_^3−^ supply, and shallow-water CaCO_3_ burial. Full details of the model configuration, parameterizations, procedures, and experimental design are provided in Material and Methods, Supplementary Text, tables S2 to S4, and code S1.

**Fig. 4.**
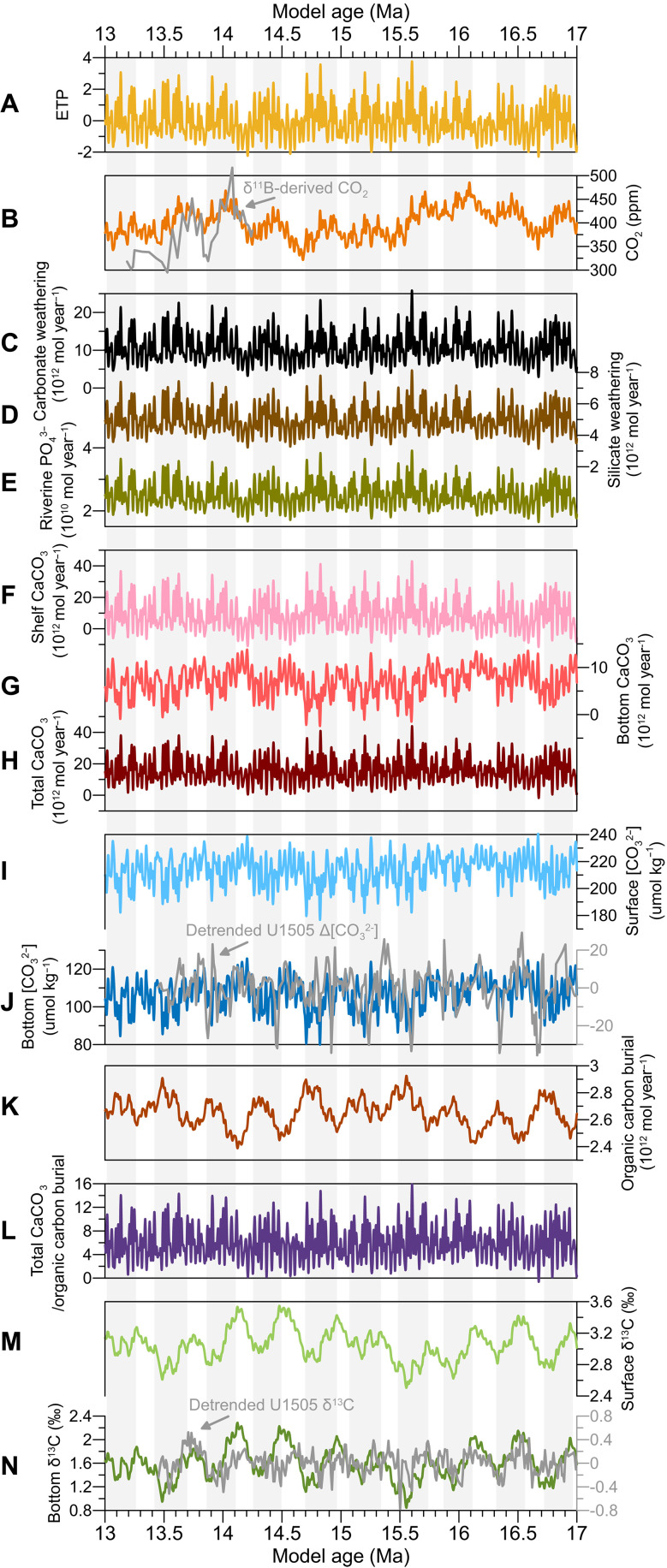
Simulated fluctuations of the marine carbon cycle on orbital timescales in the integrated experiment. (**A**) ETP (*6*). (**B**) Atmospheric CO_2_ concentrations. (**C** to **E**) Weathering rates of carbonate and silicate rocks and riverine [PO_4_^3−^] input. (**F** to **H**) CaCO_3_ burial in the shelf, basin, and total ocean. (**I** and **J**) [CO_3_^2−^] in the surface and deep ocean. (**K**) Organic carbon burial in the ocean. (**L**) Burial ratio of total CaCO_3_ to organic carbon. (**M** and **N**) δ^13^C in the surface and deep ocean (*9**,* *12*). Light gray curves represent proxy reconstructions used for comparison with model results. The comparison shows that our simulations of atmospheric CO_2_ (*42*), deep-water [CO_3_^2−^], and seawater δ^13^C are closely consistent with the reconstructed records.

The outputs of the integrated experiment display strong variability in the eccentricity bands, particularly within the 405-kyr cycle, closely tracking the ETP signal (Fig. 4). Simulated atmospheric CO_2_ remains in phase with eccentricity, and both the magnitude and pacing of its fluctuations match the proxy-based reconstructions (Fig. 4B) (*42*). Carbonate and silicate weathering fluxes, as well as riverine PO_4_^3−^ supply, also respond coherently to orbital forcing (Fig. 4, C to E). The inputs of DIC and ALK modulate shallow-water CaCO_3_ accumulation, increasing during eccentricity maxima and decreasing during minima (Fig. 4F). These CaCO_3_ burial responses thereby regulate seawater [CO_3_^2−^], which fluctuates inversely with eccentricity, with amplitudes of 30 to 40 μmol kg^−1^ (Fig. 4, I and J), consistent with the series from Site U1505 (Fig. 4J and fig. S3). The resulting [CO_3_^2−^] dynamics cause bottom-water CaCO_3_ deposition to be antiphased with that in shallow seas (Fig. 4, F and G). Because shallow-water deposition dominates the global CaCO_3_ budget, total burial exhibits a similar temporal pattern (Fig. 4, F and H). PO_4_^3−^ delivery controls surface productivity and drives particulate organic carbon (POC) sequestration in phase with eccentricity. Together, these coupled processes shape δ^13^C variability in both surface and deep waters, which varies inversely with eccentricity (Fig. 4, M and N), with amplitudes of 0.8 to 1.0‰, in agreement with the U1505 record (Fig. 4N and fig. S3).

The sensitivity experiments support the results of the integrated simulations and clarify the relative contributions of individual processes (fig. S7). Fixing riverine PO_4_^3−^ input while allowing carbonate and silicate weathering to respond to orbital forcing produces a seawater δ^13^C amplitude comparable to that of the fully coupled run (fig. S7, A and B). In contrast, holding weathering constant while permitting PO_4_^3−^ inputs to vary reduces the δ^13^C amplitude (fig. S7, C and D), demonstrating that DIC and ALK delivery is the primary driver of δ^13^C variability in the ocean, with PO_4_^3−^ supply and POC burial contributing to a lesser extent. Shallow-water CaCO_3_ dynamics further influence the ocean’s chemical response. When shelf CaCO_3_ burial is paced by the orbital cycles, it amplifies the δ^13^C signal and also governs variations in oceanic [CO_3_^2−^] (fig. S7). In the absence of riverine DIC and ALK removal through CaCO_3_ sequestration on continental shelves, modeled [CO_3_^2−^] would vary in phase with eccentricity (fig. S7, B and D), opposite to the pattern observed at Site U1505 (Fig. 4J and fig. S3).

## DISCUSSION

### Miocene marine carbonate system changes

**Fig. 5.**
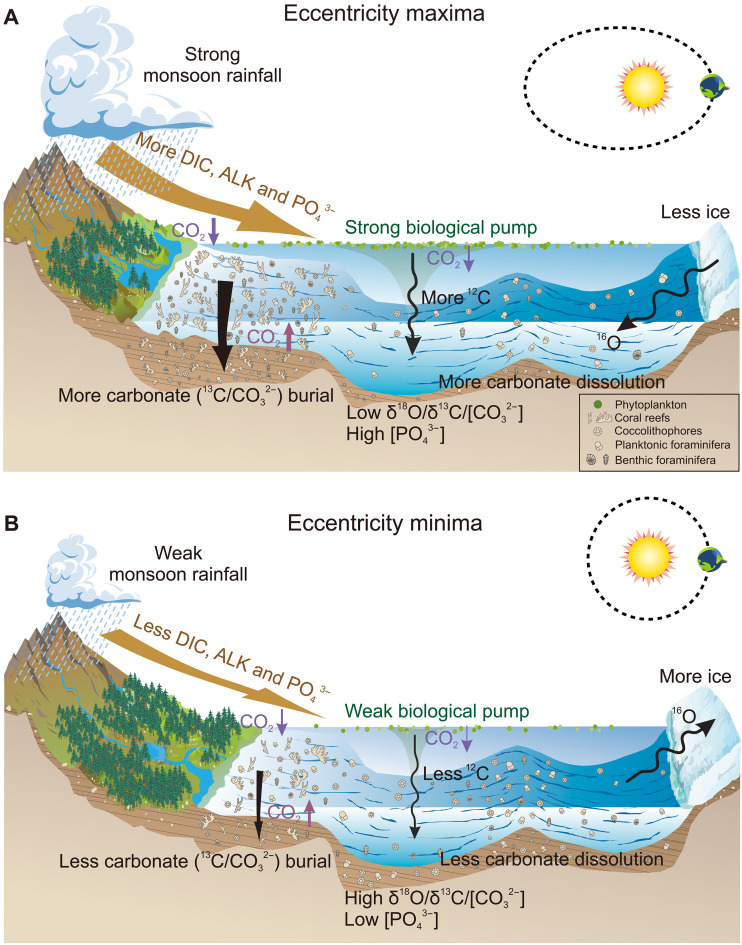
Schematic illustration of mechanisms driving continental weathering and shelf-basin carbonate partitioning, exemplified by the case when perihelion aligns with the Northern Hemisphere summer. (**A**) Eccentricity maxima caused an increase in seawater temperature and a reduction in Antarctic ice volume, leading to a decrease in deep-sea δ^18^O. Simultaneously, intensified monsoons and continental weathering transported more DIC, ALK, and PO_4_^3−^ to the ocean, resulting in increased carbonate accumulation on submerged continental shelves and enhanced marine biological pump strength. Together, these processes reduced deep-sea δ^13^C and [CO_3_^2−^] while raising atmospheric CO_2_ and [PO_4_^3−^]. (**B**) During eccentricity minima, the opposite effects occurred.

Our proxy data and modeling results suggest that tropical and subtropical chemical weathering, along with associated shifts in the marine carbonate system, can account for the synchronous δ^18^O-δ^13^C pattern on eccentricity timescales during the Miocene (Figs. 1 to 4 and figs. S4 and S7). At eccentricity maxima, a more active hydrological cycle in low latitudes, as evidenced by marine and terrestrial proxies (fig. S8, B and F) (*43*, *44*), intensified carbonate and silicate weathering and boosted riverine transport of DIC and ALK (Figs. 4 and 5A and figs. S7 and S9). These inputs, combined with Antarctic ice sheet retreat and the resulting transgression, could trigger immense carbonate deposition over the continental margins (Fig. 5A and fig. S9).

Nowadays, shelf (~35 to 45%) and basin (~55 to 65%) environments sequester similar proportions of carbonate (*45*), whereas the much narrower shallow-water zones retained only about ^1^/_10_ of the carbonate when sea level dropped by 120 m during Quaternary glacial periods (*46*). Throughout the early to mid-Miocene, global sea level was ~50-m higher than present and fluctuated by 30 to 40 m at eccentricity cycles (*47*), which is also inferred to have notably altered the accommodation space available for carbonate burial in these shallow areas (*48*). Therefore, during periods of maximum eccentricity, substantial carbonate deposition in broader shelf areas would, in turn, increase the consumption of DIC and ALK, leading to reduced seawater [CO_3_^2−^] and suppressed deep-sea carbonate accumulation globally (Figs. 2 to 4 and 5A and figs. S7 and S9). With shelf burial accounting for the majority of global carbonate accumulation, the total burial would rise in tandem. In addition, the cumulative rise in global carbonate burial promoted CO_2_ release from the ocean to the atmosphere, buffering the CO_2_ removed by rock weathering and organic carbon burial (Figs. 4 and 5A and figs. S6B and S7). This resulted in relatively higher CO_2_ levels during eccentricity maxima (Figs. 4 and 5A and fig. S7) (*42*).

Enhanced global carbonate burial, most of which occurs in shelf environments, helps explain the decline in seawater δ^13^C at eccentricity maxima (Figs. 4 and 5A and figs. S7 and S9). Biogenic carbonates preferentially incorporate ^13^C during precipitation (*49*). In particular, aragonite-forming corals, the dominant carbonate producers on continental shelves, exhibit a δ^13^C fractionation of ~2.7‰ compared with ~0.9‰ for calcite-forming organisms such as foraminifera and coccolithophores (*49*). Although our model does not explicitly differentiate between carbonate mineralogies and assumes no isotopic fractionation during carbonate formation, large-scale carbonate accumulation nonetheless drives synchronous δ^13^C depletion in both surface and deep waters (*50*) (Fig. 4 and fig. S7).

In our model, the increased delivery of riverine DIC, with an assumed δ^13^C value of −5‰ (see fig. S10 for justification), likely plays a key role in lowering seawater δ^13^C during eccentricity maxima (fig. S7, A and B). Now, global riverine DIC export is estimated at 0.52 ± 0.17 Pg year^−1^, accounting for roughly half of the total carbon flux to the ocean (*51*). The δ^13^C values of DIC in modern rivers typically range from −12 to −5‰ (fig. S10) (*52*, *53*), which are substantially more negative than the average seawater δ^13^C of ~0 to 2‰ (*49*). This contrast implies that, before the Late Miocene, more intense chemical weathering and enhanced riverine DIC transport would have been an effective mechanism in modulating the oceanic δ^13^C budget.

Changes in riverine PO_4_^3−^ input and sea surface productivity also contribute to seawater [CO_3_^2−^] and δ^13^C variations in eccentricity bands. Our [PO_4_^3−^] data and simulation results suggest that increased biological pump strength during eccentricity maxima caused greater export of POC, which underwent remineralization with increasing water depth, elevating [PO_4_^3−^] while lowering [CO_3_^2−^] and δ^13^C in the deep ocean (Figs. 4 and 5A and figs. S4; S7; S8, C and D; and S9) (*23*, *25*–*27*, *54*). Yet, primary productivity and organic burial linked to PO_4_^3−^ input were insufficient in our simulations to generate ~1‰ fluctuations in δ^13^C (fig. S7, C and D).

Part of our argument parallels the “coral reef hypothesis” (*55*–*57*), which was originally proposed to explain the Pleistocene glacial-interglacial oscillations in atmospheric CO_2_ levels but was later found to be inadequate for that purpose. Although largely disregarded for decades, this hypothesis offers a compelling framework for interpreting the eccentricity-paced variations in benthic δ^18^O and δ^13^C, seawater [CO_3_^2−^] and [PO_4_^3−^], deep-sea %CaCO_3_ burial, and atmospheric CO_2_ identified in this study (Figs. 1 to 4 and fig. S4). Its relevance during the Miocene stems from intensified chemical weathering that increased DIC and ALK inputs, the broader extent of continental shelves that provided expanded depositional space, and a substantially greater mass of carbonate burial relative to organic carbon.

### Extrapolating the interpretation to the Oligocene

Benthic δ^18^O and δ^13^C continued to stay in phase on eccentricity timescales throughout the Paleocene to Oligocene (Fig. 1, E and F) (*7*–*10*), raising the question of whether the “weathering and carbonate burial” hypothesis was also operative during the early Cenozoic warm intervals. The Paleocene-Eocene featured climatic boundary conditions distinct from those of the Miocene (Fig. 1, A and B) (*2*) and was characterized in particular by an ice-free world in which benthic δ^18^O seemed to predominantly track deep-ocean temperature rather than changes in ice volume or sea level. During this epoch, the in-phase pattern (*2*, *10*, *14*, *58*–*60*) was attributed to eccentricity-modulated precession forcing, which potentially amplified low-latitude climate processes regulating the land-ocean carbon transfer (*14*, *15*, *61*), and was possibly further complicated by episodic hyperthermal events (e.g., the Paleocene-Eocene thermal maximum) (Fig. 1, A and B), triggered by abrupt carbon injections from isotopically depleted sources (*61*–*64*). Given these confounding factors, the role of carbonate deposition during the Miocene may not be directly applicable to an ice-free regime. In contrast, the Oligocene, characterized by the onset of unipolar glaciation, shared similar climatic boundary conditions with the Miocene (Fig. 1) (*2*, *7*, *65*, *66*). Although direct reconstructions of deep-ocean [CO_3_^2−^] are lacking for this period, synchronized eccentricity-paced variations in benthic δ^18^O, δ^13^C, and deep-sea %CaCO_3_ observed in both the Pacific (*5*) and Atlantic (*22*, *24*, *41*) (fig. S11) indicate that a Miocene-like carbonate burial rhythm was probably already in place. This evidence largely supports the extension of our proposed argument to the Oligocene.

Note that nuanced differences exist between the Oligocene and Miocene (*7*). Owing to the ~65-kyr residence time of carbon in the ocean (*5*, *7*, *24*, *67*), the overall in-phase relationship between δ^18^O and δ^13^C across the Oligocene-Miocene interval was superimposed by δ^18^O leading δ^13^C by ~17 kyr in the 405-kyr band and ~1.9 to 2.5 kyr in the 100-kyr band (*7*, *9*). Nevertheless, this δ^18^O-lead-δ^13^C scenario may diminish or even reverse, producing a δ^13^C-lead-δ^18^O behavior when the carbon cycle was accelerated by events such as tectonic CO_2_ degassing during the Miocene Climate Optimum (*9*) or under extreme astronomical forcing (*7*). The prolonged ~2.4-Myr eccentricity cycle, a manifestation of the latter, was found to modulate the δ^18^O-δ^13^C phase interaction, with δ^13^C leading δ^18^O at the ~2.4-Myr minima during the Oligocene, but shifting to the maxima during the Miocene (*7*). This shift was considered to be caused by alterations in the locus of chemical weathering at the ~2.4-Myr eccentricity extremes, with greater contributions from the southern high latitudes in the Oligocene and from the low latitudes in the Miocene (*7*). Outside these special periods, tropical-subtropical chemical weathering and shelf-basin carbonate partitioning remained fundamental mechanisms responsible for exerting primary control on carbon-cycle dynamics at both the 405- and 100-kyr cycles throughout the Oligocene and Miocene.

### Post-Miocene interbasin discrepancy in the marine carbonate system

Since the Late Miocene, global benthic δ^18^O and δ^13^C records have exhibited a notable reduction in signal strength, with their relationship reversing from in-phase to antiphase on eccentricity timescales (Fig. 1) (*2*, *7*, *10*, *11*). This transition has been ascribed to the development of a bipolar glaciated world (*7*, *10*, *11*). During eccentricity minima, the expansion of ice caps in the Northern Hemisphere likely led to a contraction of the terrestrial biosphere and enhanced the strength of the marine biological pump due to increased inland aridification and a rise in dust fluxes into the ocean. Both processes facilitated a net transfer of ^12^C from land to sea, resulting in lower δ^13^C values (*7*, *10*, *11*). Meanwhile, unlike the Miocene, changes in the marine carbonate system during the Pliocene-Pleistocene appeared to be regionally variable rather than globally consistent, especially after the further intensification of Northern Hemisphere glaciation at ~2.7 Ma. According to existing reconstructions, benthic δ^18^O variations were in phase with Δ[CO_3_^2−^] oscillations and the deep-sea %CaCO_3_ burial at 405- and 100-kyr cycles in the Pacific but displayed a nearly antiphase behavior in the Atlantic (figs. S12 to S14) (*22*, *24*, *25*, *27*, *41*, *68*–*78*). This interbasin discrepancy may manifest as a “seesaw” pattern on eccentricity-band fluctuations, with its counteracting effect potentially weakening the ability of the oceanic carbonate system to regulate global carbon cycling. A more comprehensive understanding of this complexity will require future model-data integration.

## MATERIALS AND METHODS

### Measurement of benthic stable isotopes

Samples were oven-dried at 60°C, weighed, soaked in water for 48 hours to disaggregate, and washed over a 63-μm sieve. Residues were dried again at 60°C, weighed, and sieved into >150-μm size fraction. Well-preserved specimens of epibenthic *Cibicidoides wuellerstorfi* or *C. mundulus* were picked, crushed into large fragments, and cleaned in alcohol using an ultrasonic bath. Stable isotopes were measured at the State Key Laboratory of Marine Geology, Tongji University, with a Thermo-Finnigan MAT 253 mass spectrometer coupled online to a CarboKiel Device (Type IV), where individual samples were reacted with 99% H_3_PO_4_ at 70°C. The long-term analytical precision (1σ) is better than ±0.07‰ for δ^18^O and ±0.04‰ for δ^13^C. Results were calibrated against National Bureau of Standards 19 and reported in per mil (‰) relative to Vienna Pee Dee Belemnite. *Cibicidoides* δ^18^O values were adjusted by +0.64‰.

### Chronology of Site U1505

An astronomically tuned age model for Site U1505, covering the period between 21 and 6 Ma, was previously published (*9*) by correlating its benthic foraminiferal δ^18^O series with those of Sites 1264 (21 to 17 Ma) (*22*, *24*), U1337 (20 to 17 Ma) (*35*), and 1146 (17.4 to 6 Ma) (*43*). The chronologies for these reference sites were constructed by linking their respective benthic δ^18^O records to orbital solutions, with support from biostratigraphy and magnetostratigraphy. In this study (fig. S2), we refined the 24.3- to 21-Ma chronology for Site U1505 by aligning its benthic δ^18^O data with eccentricity (*6*) using the “QAnalyseries 2.0.8” software (*79*, *80*). Before this alignment, a preliminary framework was developed on the basis of planktonic foraminiferal and calcareous nannofossil datums (*17*), enabling us to identify key δ^18^O and δ^13^C markers, including the Oligocene-Miocene transition (380.8 m to 23.1 Ma) and the Early Miocene carbon shift (356.1 m to 21.8 Ma).

### Trace element analyses

Well-preserved specimens of *C. mundulus*, with shell sizes of 250 to 500 μm (>8 specimens) or 500 to 850 μm (2 to 6 specimens) (fig. S15), were selected for the trace element analysis. Adjacent samples were combined to obtain sufficient foraminiferal material for analysis where necessary. The shells were cracked between two glass slides and cleaned using oxidative-reductive procedures (*81*, *82*) to remove clay particles, Fe-Mn oxides, authigenic Mn carbonate, and organic matter. After cleaning, they were inspected under a microscope to exclude inadequately processed shells and residual contaminants.

Trace element concentrations were measured using an inductively coupled plasma mass spectrometer (VG-X7) at the State Key Laboratory of Marine Geology, Tongji University. To monitor the long-term reproducibility of the instrument, 142 standards were analyzed for the 24.3- to 20.9-Ma interval, resulting in external reproducibilities (1σ) of 0.20 μmol mol^−1^ for B/Ca and 0.004 μmol mol^−1^ for Cd/Ca. For the 17.4- to 13.5-Ma interval, 144 standards were tested, yielding external reproducibilities of 0.28 μmol mol^−1^ for B/Ca and 0.004 μmol mol^−1^ for Cd/Ca. Measurement precision (1σ) for B/Ca and Cd/Ca in *C. mundulus* specimens, derived from 9 replicates for the 24.3 to 20.9 Ma interval and 10 replicates for the 17.4- to 13.5-Ma interval, was 2.46 and 1.77 μmol mol^−1^ for B/Ca and 0.007 and 0.004 μmol mol^−1^ for Cd/Ca, respectively.

### Correction for shell size

To assess the effect of shell size on B/Ca and Cd/Ca ratios, we analyzed 74 paired samples of *C. mundulus* in the size ranges of 250 to 500 μm and 500 to 850 μm (fig. S15). These paired samples were collected from the same layers at various depths. The B/Ca measurements were highly consistent, with 1σ of 4.46 μmol mol^−1^, suggesting a negligible influence of shell size on B/Ca values. To account for the minor size effect on B/Ca values, we applied a linear regression with the intercept fixed at zero (fig. S16). In contrast, Cd/Ca ratios displayed more variability between the two shell size ranges, with 1σ of 0.024 μmol mol^−1^. Specimens in the 250- to 500-μm range consistently exhibited higher Cd/Ca ratios than those in the 500- to 850-μm range, with an average offset of 0.039 μmol mol^−1^. To address this discrepancy, a similar linear regression was applied to adjust for the impact of shell size on Cd/Ca results (fig. S16).

### Converting B/Ca to ∆[CO_3_^2−^]

B/Ca ratios of *C. mundulus* (250 to 500 μm) from global core-top samples show a linear calibration with deep-water Δ[CO_3_^2−^]: B/Ca = (0.69 ± 0.04) × Δ[CO_3_^2−^] + (119.9 ± 1.7) (*29*, *83*–*86*). The residence time of B in seawater is ~14 to 20 Ma (*87*–*90*), while that of Ca is ~1 Ma (*91*). Given that this study covers the period from 24.3 to 20.9 Ma and 17.4 to 13.4 Ma, it is essential to account for historical variations in seawater B/Ca. Recent experiments, however, have revealed that [Ca] does not affect B/Ca in planktonic foraminiferal shells (*92*). Thus, we corrected only for past changes in seawater [B] when adjusting B/Ca values.

### Converting Cd/Ca to [PO_4_^3−^]

The Cd/Ca ratios in benthic foraminifera (Cd/Ca)_benthic_ reflect the concentration of dissolved Cd in seawater ([Cd]_sw_), with its distribution coefficient (*D*_Cd_) defined as follows (*30*, *93*)

$$D_{\text{Cd}}={\left(\text{Cd}/\text{Ca}\right)}_{\text{benthic}}/{\left(\text{Cd}/\text{Ca}\right)}_{\text{sw}}$$

where (Cd/Ca)_sw_ denotes the Cd/Ca ratios in seawater. The value of *D*_Cd_ varies primarily with water depth (*30*, *93*)

$$D_{\text{Cd}}=1.3,\text{depth}<1150\;m$$

$$D_{\text{Cd}}=1.3+\left(\text{depth}-1150\right)\times 1.6/1850,1150<\text{depth}<3000\;m$$

$$D_{\text{Cd}}=2.9,\text{depth}>3000\;m$$

The water depth at Site U1505 is 2916.6 m, corresponding to a calculated *D*_Cd_ of 2.83. In the modern ocean, [Cd]_sw_ and [PO_4_^3−^] exhibit a nonlinear relationship (*93*–*95*). We compiled global [Cd]_sw_ and [PO_4_^3−^] data from the GEOTRACES 2021 dataset (*96*, *97*) to establish the following relationship for reconstructing past deep-sea [PO_4_^3−^] (fig. S17)

$$\begin{array}{c} \left[{\text{PO}}_{4}^{3-}\right]=\left(2.790\pm 0.018\right)\times \\ \sqrt{\left[\text{Cd}\right]\text{sw}}+\left(0.116\pm 0.015\right)\;\left(\text{Pacific Ocean},\text{used in this study}\right) \end{array}$$

$$\begin{array}{c} \left[{\text{PO}}_{4}^{3-}\right]=\left(2.858\pm 0.011\right)\times \\ \sqrt{\left[\text{Cd}\right]\text{sw}}+\left(0.142\pm 0.007\right)\;\left(\text{Global Ocean}\right) \end{array}$$

### Uncertainty estimation

We reported uncertainties for B/Ca-derived Δ[CO_3_^2−^] and Cd/Ca-based [PO_4_^3−^] as 2σ, incorporating analytical and calibration components. The analytical uncertainties, determined by the measurement precision of B/Ca (±2.46 μmol mol^−1^ for 24.3 to 20.9 Ma and ± 1.77 μmol mol^−1^ for 17.4 to 13.5 Ma) and Cd/Ca (±0.007 μmol mol^−1^ for 24.3 to 20.9 Ma and ± 0.004 μmol mol^−1^ for 17.4 to 13.5 Ma), were used to generate 2000 sequences for each element ratio, modeled as normally distributed independent variables. The calibration uncertainties, defined by the slope (0.69 ± 0.04 μmol mol^−1^ for Δ[CO_3_^2−^] and 2.790 ± 0.018 μmol mol^−1^ for [PO_4_^3−^]) and intercept (119.9 ± 1.7 μmol mol^−1^ Δ[CO_3_^2−^] and 0.116 ± 0.015 μmol mol^−1^ for [PO_4_^3−^]), were similarly addressed by constructing 2000 normally distributed pairs of slopes and intercepts. These surrogate values were then applied to the perturbed B/Ca and Cd/Ca data for calculating the corresponding Δ[CO_3_^2−^] and [PO_4_^3−^].

### Time-frequency analysis

We conducted multitaper spectral analysis, evolutionary multitaper spectral analysis, and Gaussian bandpass filtering using Acycle 2.8 (*98*). Before analysis, the records were interpolated into evenly spaced sequences with a step size equal to the original data resolution. Long-term trends (periods longer than 1 Myr) were then removed using the locally weighted regression method. The data were filtered within the frequency ranges of 0.0022222 to 0.0027778 (for 405 kyr) and 0.0074074 to 0.011111 (for 100 kyr).

We performed cross-spectral fast Fourier transform analysis to calculate the evolutionary phase relationship between proxies, as detailed in the (*7*). The cross-spectra were computed using a 1.2-Myr-wide window with a 0.2-Myr step, focusing on the 405-kyr (360 and 450 kyr) and 100-kyr (90 and 135 kyr) frequency bands to extract the phase relationship. Coherence values of 0.3 and 0.6 correspond to confidence levels of ~70 and ~97.5%, respectively, of nonzero coherence.

### Model configuration and experimental design

Our model (fig. S6A) (*9*, *12*) comprises an atmosphere box “A”; three surface ocean boxes “S,” “E,” and “N”; two deep ocean boxes “D” and “I”; and a bottom ocean box “B.” The transfer of CO_2_ between the atmosphere and the surface ocean is expressed as “*g_ij_*”, where *i* and *j* refer to the atmosphere and individual surface boxes, respectively. The flow *Q*_1_ corresponds to the northern component water, whereas *Q*_2_ and *Q*_3_ represent the southern component water masses, together resembling modern ocean circulation patterns. Mixing between adjacent boxes is described by “*f_ij_*”, where *i* and *j* denote any pair of neighboring boxes. The export production of POC from the surface ocean (“*p_s_*”, “*p_e_*”, and “*p_n_*”) is transferred to deeper waters, where a fraction “*g”* undergoes remineralization within the deep and bottom ocean. Only a minor portion of this POC is ultimately preserved in sediments. CaCO_3_ is buried in both the equatorial surface ocean and deep-sea sediments.

The model incorporates key biogeochemical processes (fig. S6B), including atmospheric CO_2_ inputs from volcanic degassing and the oxidation of sedimentary organic carbon; chemical weathering of carbonate and silicate rocks; riverine delivery of DIC, ALK, and PO_4_^3−^; marine primary productivity; CaCO_3_ precipitation and dissolution; and organic carbon burial.

The weathering rates of carbonate (*W*_car_) and silicate (*W*_sil_) rocks are parameterized as functions of atmospheric CO_2_ concentration

$$W_{\text{car}}=f_{\text{car}}\times \left(p{\text{CO}}_{2}/p{\text{CO}}_{2,\text{ref}}\right)$$

$$W_{\text{sil}}=f_{\text{sil}}\times {\left(p{\text{CO}}_{2}/p{\text{CO}}_{2,\text{ref}}\right)}^{\mathrm{\alpha s}}$$

Here, *f*_car_ (10.7 × 10^12^ mol year^−1^) and *f*_sil_ (5 × 10^12^ mol year^−1^) represent the baseline fluxes of carbonate and silicate weathering, respectively. *p*CO_2_ denotes the partial pressure of atmospheric CO_2_, and *p*CO_2,ref_ is fixed at 400 parts per million, representing conditions during the Miocene Climate Optimum. The weathering sensitivity constant (α_s_) is assigned a value of 0.3.

CO_2_ remains in the atmosphere box A, whereas riverine DIC and ALK are supplied to the equatorial surface box E at a flux ratio of *W*_car_: 2 × (*W*_sil_ + *W*_car_). The riverine PO_4_^3−^ input (rivPO_4_) is linked to silicate weathering and is given by:

$${\text{rivPO}}_{4}=W_{\text{sil}}/200$$

Surface primary production in the high-latitude boxes S and N is prescribed. In the low-latitude box E, POC export is regulated by [PO_4_^3−^], which is almost entirely used by phytoplankton supplied via riverine input and lateral mixing. The fraction of primary production ultimately buried as organic carbon in the deep ocean is held constant at 1%. Shallow-water CaCO_3_ sequestration is maintained at 9 × 10^12^ mol year^−1^. Deep-water CaCO_3_ burial is controlled by deep-sea [CO_3_^2−^], with deposition occurring when [CO_3_^2−^] exceeds 85 μmol kg^−1^ and dissolution when it falls below this threshold.

The δ^13^C composition of CO_2_ released by volcanic degassing and the oxidation of sedimentary organic matter is held constant at −5‰. The δ^13^C of riverine carbon input is set to −5‰ (fig. S10). Isotopic fractionation associated with algal POC formation is assigned ε_p_ = −23‰, while that during carbonate precipitation is neglected under the assumption that particulate inorganic carbon is in isotopic equilibrium with ambient seawater.

The model is run from its initial state for 2 Myr until it reaches equilibrium then forced by orbital variations expressed as ETP (*6*) and extended for another 4 Myr. We conducted an integrated experiment to capture the overall response of the system, accompanied by four sensitivity experiments designed to separate the effects of riverine DIC-ALK fluxes, PO_4_^3−^ delivery, and shallow-water CaCO_3_ burial. The experimental framework is outlined below:

Integrated experiment: The baseline fluxes of carbonate and silicate weathering, along with shallow-water CaCO_3_ burial, are coupled to orbital forcing, with *f*_car_ = 10.7 × 10^12^ × (1 + 0.3 × ETP) mol year^−1^, *f*_sil_ = 5.0 × 10^12^ × (1 + 0.15 × ETP) mol year^−1^, and Carb_sh_ = 9 × 10^12^ × (1 + ETP) mol year^−1^.

Experiment 1: This simulation follows the integrated setup but maintains a constant riverine PO_4_^3−^ supply (rivPO_4_ = 2.5 × 10^10^ mol year^−1^).

Experiment 2: Building on Experiment 1, shallow-water CaCO_3_ burial is kept steady at Carb_sh_ = 9 × 10^12^ mol year^−1^.

Experiment 3: Here, the baseline fluxes of carbonate and silicate are prescribed as constant values (*f*_car_ = 10.7 × 10^12^ mol year^−1^, *f*_sil_ = 5.0 × 10^12^ mol year^−1^), while PO_4_^3−^ delivery continues to vary with orbital forcing and is represented as rivPO_4_ = 5.0 × 10^10^ × (1 + 0.15 × ETP) mol year^−1^. Shallow-water CaCO_3_ burial remains responsive to orbital changes.

Experiment 4: Following the configuration of experiment 3, shallow-water CaCO_3_ burial is instead imposed as a constant parameter.

Readers interested in a more detailed description of the model structure, assumptions, implementation, and experimental setup are referred to the Supplementary Text, tables S2 to S4, and the code S1 provided with this study.

## Notes

**Correction (18 June 2026):** Due to a production error, “*D*_Cd_ = 2.9, depth > 3000 m” was incorrectly written as “*D*_Cd_ = 1.3, depth > 3000 m” in the Materials and Methods section. The equation has been corrected.

## Supplementary Materials

### The PDF file includes:

Provenance Supplementary Text Figs. S1 to S17 Tables S1 to S4 Legends for data S1 to S4 Legend for code S1 References

### Other Supplementary Material for this manuscript includes the following:

Data S1 to S4

Code S1
