# Supplementary material for: Eccentricity rhythms in the Oligocene-Miocene carbon cycle regulated by weathering and carbonate burial
Source: Sci Adv. 2026 Jan 30;12(5):eadx6682. doi: 10.1126/sciadv.adx6682 (PMC12857679; doi:10.1126/sciadv.adx6682)
Supplement: Supplementary file 1 — Provenance Supplementary Text Figs. S1 to S17 Tables S1 to S4 Legends for data S1 to S4 Legend for code S1 References [file sciadv.adx6682_sm.pdf]

Science Advances
AAAS

Supplementary Materials for

Eccentricity rhythms in the Oligocene-Miocene carbon cycle regulated by weathering and carbonate burial

Fenghao Liu *et al.*

Corresponding author: Enqing Huang, ehuang@tongji.edu.cn

Sci. Adv. **12**, eadx6682 (2026)
DOI: 10.1126/sciadv.adx6682

The PDF file includes:

Provenance
Supplementary Text
Figs. S1 to S17
Tables S1 to S4
Legends for data S1 to S4
Legend for code S1
References

Other Supplementary Material for this manuscript includes the following:

Data S1 to S4
Code S1

Provenance

The sediment samples used in this study were collected from Site U1505 during the International Ocean Discovery Program (IODP; https://www.iodp.org) Expedition 368 in the South China Sea in 2017 by the IODP Science Party, following standard ocean-drilling protocols. The lithostratigraphy, biostratigraphy, paleomagnetism, geochemistry, physical properties, and downhole measurements for this site were established by the Expedition 368 shipboard scientific team, as documented in the official shipboard report (https://publications.iodp.org/preliminary_report/378). Following recovery, all samples were archived at the IODP Kochi Core Center (KCC; https://www.kochi-core.jp/en), where they are publicly accessible under the IODP open-access sampling policy. The subsamples analyzed here were formally requested from KCC, and all remaining processed materials, including the foraminiferal specimens, are currently curated at the State Key Laboratory of Marine Geology, Tongji University.

Supplementary Text

Model framework and experimental design

1. Physical scheme

The model consists of one atmosphere box and six ocean boxes (Fig. S6A). The surface ocean is subdivided at 45°S and 45°N into three regions: the southern box “S”, the equatorial box “E”, and the northern box “N”, each extending to a depth of 200 m. Beneath the surface, the ocean interior is represented by a northern deep box “D” (to 2500 m), a southern intermediate box “I” (to 2500 m), and a bottom box “B” (to 4000 m). Air-sea CO_2 exchange occurs through the fluxes “g_{as}”, “g_{ae}”, and “g_{an}”. The overturning circulation involves two main pathways. Q_1 describes the Northern Component Water, analogous to modern North Atlantic Deep Water, while Q_2 and Q_3 together represent the Southern Component Water, comparable to Antarctic Bottom Water. Mixing between neighboring boxes is described by fluxes “f_{si}”, “f_{ib}”, “f_{ed}”, “f_{db}”, “f_{nd}”, “f_{se}”, “f_{en}”, and “f_{id}”. The configuration of boxes and their associated flow parameters are listed in Table S2. Surface boxes export POC and PIC via the fluxes “p_s”, “p_e”, and “p_n”. These particles sink into deeper layers, where remineralization or dissolution (“g”) takes place within boxes “I”, “D”, and “B”. Only a small fraction of the sinking POC is ultimately buried in deep-sea sediments. $CaCO_3$ burial is partitioned into shallow- and deep-water components, which are removed from boxes “E” and “B”, respectively.

2. Biogeochemical scheme

The model incorporates key biogeochemical processes (Fig. S6B), including volcanic degassing and sedimentary oxidation that release CO_2, weathering of carbonates and silicates, riverine inputs of DIC, ALK, and PO_4^{3-}, surface primary productivity, the precipitation and dissolution of $CaCO_3$, and burial of organic matter. Detailed descriptions of each process are provided in the following sections, and the corresponding parameter values are summarized in Table S3.

2.1 Atmospheric CO_2

The atmospheric box receives CO_2 released from volcanic degassing and sedimentary oxidation ($DG_{volkero}$) and exchanges CO_2 with the three surface ocean boxes, as formulated below:

$$\frac{dp\mathrm{CO}_{2,\mathrm{A}}}{dt} = \frac{1}{V_{atm}} \times [\underbrace{sol_S \times (p\mathrm{CO}_{2,\mathrm{S}} - p\mathrm{CO}_{2,\mathrm{A}})}_{\text{air-sea exchange}} + \underbrace{sol_E \times (p\mathrm{CO}_{2,\mathrm{E}} - p\mathrm{CO}_{2,\mathrm{A}})}_{\text{air-sea exchange}} + \underbrace{sol_N \times (p\mathrm{CO}_{2,\mathrm{N}} - p\mathrm{CO}_{2,\mathrm{A}})}_{\text{air-sea exchange}} + \underbrace{DG_{volkero}}_{\text{volcanic degassing and kerogen oxidation}}] \quad \text{(Eq. S1)}$$

Air-sea CO_2 exchange in the surface ocean is governed by the solubility of CO_2 (*sol*) and the concentration gradient between seawater and the atmosphere. CO_2 flux occurs from the ocean to the atmosphere when seawater is supersaturated with respect to atmospheric pCO_2, and from the atmosphere to the ocean when seawater is undersaturated.

2.2 Weathering

The weathering rates of carbonate and silicate rocks are parameterized as functions of the atmospheric CO_2 concentration(*99*):

$$W_{car} = f_{car} \times (p\mathrm{CO}_2 / p\mathrm{CO}_{2,\mathrm{ref}}) \text{ (Eq. S2)}$$

$$W_{sil} = f_{sil} \times (p\mathrm{CO}_2 / p\mathrm{CO}_{2,\mathrm{ref}})^{\alpha_s} \text{ (Eq. S3)}$$

where W_{car} and W_{sil} represent the weathering rates of carbonate and silicate rocks, respectively, f_{car} and f_{sil} denote their baseline values, pCO_2 is the atmospheric partial pressure of CO_2, $pCO_{2,\mathrm{ref}}$ is the reference value, and α_s is the weathering sensitivity constant. Riverine DIC and ALK fluxes are governed by these weathering rates through the following reactions:

$$\mathrm{CaCO_3 + CO_2 + H_2O \rightarrow Ca^{2+} + 2HCO_3^-} \text{ (Eq. S4)}$$

$$\mathrm{CaSiO_3 + 2CO_2 + H_2O \rightarrow Ca^{2+} + 2HCO_3^- + SiO_2} \text{ (Eq. S5)}$$

Carbonate weathering consumes one mole of CO_2 and releases two moles of DIC to the ocean, producing a net ΔDIC : ΔALK ratio of 1:2 in the atmosphere-ocean system(*100*). In contrast, silicate weathering consumes two moles of CO_2 from the atmosphere and releases two moles of DIC, resulting in no net change in DIC but contributing two moles of ALK.

Here, CO_2 is not explicitly transferred from the atmospheric box to the surface ocean box receiving riverine input, as the two reservoirs equilibrate rapidly(*99*). Instead, CO_2 remains in the atmosphere, while riverine DIC and ALK are supplied to the surface box "E" in the ratio $W_{\mathrm{car}} : 2 \times (W_{\mathrm{sil}} + W_{\mathrm{car}})$. This simplification introduces negligible errors in atmospheric CO_2 concentration and surface ocean carbonate chemistry.

Additionally, riverine PO_4^{3-} flux ($rivPO_4$) is coupled to silicate weathering following:

$$rivPO_4 = W_{sil} / 200 \text{ (Eq. S6)}$$

2.3 Surface primary production

Surface primary productivities (PP_S and PP_N) in the high-latitude boxes "S" and "N" is prescribed as constant values. In the low-latitude box "E", export production (PP_E) of POC

is regulated by [PO_4^{3-}]. PO_4^{3-} supplied through riverine input and by mixing from adjacent boxes is almost entirely consumed by phytoplankton within this box(*101*, *102*).

2.4 $CaCO_3$ burial and dissolution

The burial of shallow-water $CaCO_3$ ($Carb_{sh}$), mainly derived from coral reefs and calcareous shells produced by plankton in surface waters, is prescribed as a fixed flux in the equatorial box "E". Deep-water $CaCO_3$ sedimentation in the bottom box "B" ($Carb_{bottom}$) is parameterized using the following formulation:

$$Carb_{bottom} = WDAMP \times [\mathrm{DIC}]_B / CDTARG \times ([CO_3^{2-}]_B - CDTARG) \times Vol_B \quad \text{(Eq. S7)}$$

When [CO_3^{2-}] in box "B" ([CO_3^{2-}]$_B$) exceeds the target concentration (*CDTARG*), $CaCO_3$ burial occurs. Conversely, when [CO_3^{2-}]$_B$ falls below *CDTARG*, $CaCO_3$ dissolution takes place. *WDAMP* represents the damping rate, corresponding to variations in the lysocline depth(*103*), while [DIC]$_B$ and Vol_B denote the DIC concentration and volume of box "B", respectively.

2.5 Organic matter burial

POC produced by surface primary productivity sinks through the water column and undergoes remineralization, with a fraction *g* being decomposed within the intermediate "I", deep "D", and bottom "B" boxes. Ultimately, only a fraction, denoted as *rom*, of the POC escapes remineralization and is buried in sediments within the bottom box "B".

2.6 Oceanic budgets of DIC, ALK, and PO_4^{3-}

In addition to riverine input, the concentrations of DIC, ALK, and PO_4^{3-} in each ocean box are regulated by internal biogeochemical fluxes, as formulated below:

$$\frac{d[\mathrm{DIC}]_S}{dt} = \frac{1}{Vol_S} \times [\underbrace{(f_{se}+Q_3)\times([\mathrm{DIC}]_E-[\mathrm{DIC}]_S)+\cdots}_{\text{ocean mixing}}$$
$$\underbrace{-r_{Corg:p}\times PP_S}_{\text{POC production}} \underbrace{-\,rain\ ratio\times r_{Corg:p}\times PP_S}_{\text{PIC production}} + \underbrace{sol_S\times(p\mathrm{CO}_{2,\mathrm{A}}-p\mathrm{CO}_{2,\mathrm{S}})}_{\text{air-sea exchange}}] \quad \text{(Eq. S8)}$$

$$\frac{d[\mathrm{DIC}]_E}{dt} = \frac{1}{Vol_E} \times [\underbrace{f_{se}\times([\mathrm{DIC}]_S-[\mathrm{DIC}]_E)+\cdots}_{\text{ocean mixing}}$$
$$\underbrace{-r_{Corg:p}\times PP_E}_{\text{POC production}} \underbrace{-\,rain\ ratio\times r_{Corg:p}\times PP_E}_{\text{PIC production}} + \underbrace{sol_E\times(p\mathrm{CO}_{2,\mathrm{A}}-p\mathrm{CO}_{2,\mathrm{E}})}_{\text{air-sea exchange}} \quad \text{(Eq. S9)}$$
$$- \underbrace{Carb_{sh}}_{\text{shallow-water carbonate burial}} + \underbrace{W_{car}}_{\text{riverine input}}\ ]$$

$$\frac{d[\mathrm{DIC}]_N}{dt} = \frac{1}{Vol_N} \times [\underbrace{(f_{en}+Q_1)\times([\mathrm{DIC}]_E-[\mathrm{DIC}]_N)+\cdots}_{\text{ocean mixing}}$$
$$\underbrace{-r_{Corg:p}\times PP_N}_{\text{POC production}} \underbrace{-\,rain\ ratio\times r_{Corg:p}\times PP_N}_{\text{PIC production}} + \underbrace{sol_N\times(p\mathrm{CO}_{2,\mathrm{A}}-p\mathrm{CO}_{2,\mathrm{N}})}_{\text{air-sea exchange}}] \quad \text{(Eq. S10)}$$

$$\frac{d[\mathrm{DIC}]_\mathrm{I}}{dt}=\frac{1}{Vol_I}\times[\underbrace{(f_{si}+Q_3)\times([\mathrm{DIC}]_\mathrm{S}\text{-}[\mathrm{DIC}]_\mathrm{I})+\cdots}_{\text{ocean mixing}}$$
$$+\underbrace{g\times(1\text{-}rom)\times r_{Corg:p}\times PP_S}_{\text{POC remineralization}}+\underbrace{g\times rain\ ratio\times r_{Corg:p}\times PP_S}_{\text{PIC dissolution}}] \quad \text{(Eq. S11)}$$

$$\frac{d[\mathrm{DIC}]_\mathrm{D}}{dt}=\frac{1}{Vol_D}\times[\underbrace{f_{ed}\times([\mathrm{DIC}]_\mathrm{E}\text{-}[\mathrm{DIC}]_\mathrm{D})+\cdots}_{\text{ocean mixing}}$$
$$+\underbrace{g\times(1\text{-}rom)\times r_{Corg:p}\times(PP_E+PP_N)}_{\text{POC remineralization}} \quad \text{(Eq. S12)}$$
$$+\underbrace{g\times rain\ ratio\times r_{Corg:p}\times(PP_E+PP_N)}_{\text{PIC dissolution}}]$$

$$\frac{d[\mathrm{DIC}]_\mathrm{B}}{dt}=\frac{1}{Vol_B}\times[\underbrace{f_{db}\times([\mathrm{DIC}]_\mathrm{D}\text{-}[\mathrm{DIC}]_\mathrm{B})+\cdots}_{\text{ocean mixing}}$$
$$+\underbrace{(1\text{-}g)\times(1\text{-}rom)\times r_{Corg:p}\times(PP_E+PP_N+PP_S)}_{\text{POC remineralization}} \quad \text{(Eq. S13)}$$
$$+\underbrace{(1\text{-}g)\times rain\ ratio\times r_{Corg:p}\times(PP_E+PP_N+PP_S)}_{\text{PIC dissolution}}-\underbrace{Carb_{bottom}}_{\text{bottom-water carbonate burial}}]$$

$$\frac{d[\mathrm{ALK}]_\mathrm{S}}{dt}=\frac{1}{Vol_S}\times[\underbrace{(f_{se}+Q_3)\times([\mathrm{ALK}]_\mathrm{E}\text{-}[\mathrm{ALK}]_\mathrm{S})+\cdots}_{\text{ocean mixing}}$$
$$+\underbrace{0.7\times r_{N:P}\times PP_S}_{\text{nitrate uptake}}-\underbrace{2\times rain\ ratio\times r_{Corg:p}\times PP_S}_{\text{PIC production}}] \quad \text{(Eq. S14)}$$

$$\frac{d[\mathrm{ALK}]_\mathrm{E}}{dt}=\frac{1}{Vol_E}\times[\underbrace{f_{se}\times([\mathrm{ALK}]_\mathrm{S}\text{-}[\mathrm{ALK}]_\mathrm{E})+\cdots}_{\text{ocean mixing}}$$
$$+\underbrace{0.7\times r_{N:P}\times PP_E}_{\text{nitrate uptake}}-\underbrace{2\times rain\ ratio\times r_{Corg:p}\times PP_E}_{\text{PIC production}} \quad \text{(Eq. S15)}$$
$$-\underbrace{2\times Carb_{sh}}_{\text{shallow-water carbonate burial}}+\underbrace{2\times(W_{car}+W_{sil})}_{\text{riverine input}}]$$

$$\frac{d[\mathrm{ALK}]_\mathrm{N}}{dt}=\frac{1}{Vol_N}\times[\underbrace{(f_{en}+Q_1)\times([\mathrm{ALK}]_\mathrm{E}\text{-}[\mathrm{ALK}]_\mathrm{N})+\cdots}_{\text{ocean mixing}}$$
$$+\underbrace{0.7\times r_{N:P}\times PP_N}_{\text{nitrate uptake}}-\underbrace{2\times rain\ ratio\times r_{Corg:p}\times PP_N}_{\text{PIC production}}] \quad \text{(Eq. S16)}$$

$$\frac{d[\mathrm{ALK}]_\mathrm{I}}{dt}=\frac{1}{Vol_I}\times[\underbrace{(f_{si}+Q_3)\times([\mathrm{ALK}]_\mathrm{S}-[\mathrm{ALK}]_\mathrm{I})+\cdots}_{\text{ocean mixing}} - \underbrace{0.7\times g\times(1-rom)\times r_{N:P}\times PP_S}_{\text{nitrification}} + \underbrace{2\times g\times rain\ ratio\times r_{Corg:p}\times PP_S}_{\text{PIC dissolution}}] \quad \text{(Eq. S17)}$$

$$\frac{d[\mathrm{ALK}]_\mathrm{D}}{dt}=\frac{1}{Vol_D}\times[\underbrace{f_{ed}\times([\mathrm{ALK}]_\mathrm{E}-[\mathrm{ALK}]_\mathrm{D})+\cdots}_{\text{ocean mixing}} - \underbrace{0.7\times g\times(1-rom)\times r_{N:P}\times(PP_E+PP_N)}_{\text{nitrification}} + \underbrace{2\times g\times rain\ ratio\times r_{Corg:p}\times(PP_E+PP_N)}_{\text{PIC dissolution}}] \quad \text{(Eq. S18)}$$

$$\frac{d[\mathrm{ALK}]_\mathrm{B}}{dt}=\frac{1}{Vol_B}\times[\underbrace{f_{db}\times([\mathrm{ALK}]_\mathrm{D}-[\mathrm{ALK}]_\mathrm{B})+\cdots}_{\text{ocean mixing}} - \underbrace{0.7\times(1-g)\times(1-rom)\times r_{N:P}\times(PP_E+PP_N+PP_S)}_{\text{nitrification}} + \underbrace{2\times(1-g)\times rain\ ratio\times r_{Corg:p}\times(PP_E+PP_N+PP_S)}_{\text{PIC dissolution}} - \underbrace{2\times Carb_{bottom}}_{\text{bottom-water carbonate burial}}] \quad \text{(Eq. S19)}$$

$$\frac{d[\mathrm{PO_4^{3-}}]_\mathrm{S}}{dt}=\frac{1}{Vol_S}\times[\underbrace{(f_{se}+Q_3)\times([\mathrm{PO_4^{3-}}]_\mathrm{E}-[\mathrm{PO_4^{3-}}]_\mathrm{S})+f_{si}\times([\mathrm{PO_4^{3-}}]_\mathrm{I}-[\mathrm{PO_4^{3-}}]_\mathrm{S})}_{\text{ocean mixing}} - \underbrace{PP_S}_{\text{primary production}}] \quad \text{(Eq. S20)}$$

$$\frac{d[\mathrm{PO_4^{3-}}]_\mathrm{E}}{dt}=\frac{1}{Vol_E}\times[\underbrace{f_{se}\times([\mathrm{PO_4^{3-}}]_\mathrm{S}-[\mathrm{PO_4^{3-}}]_\mathrm{E})+f_{en}\times([\mathrm{PO_4^{3-}}]_\mathrm{N}-[\mathrm{PO_4^{3-}}]_\mathrm{E})}_{\text{ocean mixing}} + \underbrace{(Q_1+Q_3+f_{ed})\times([\mathrm{PO_4^{3-}}]_\mathrm{D}-[\mathrm{PO_4^{3-}}]_\mathrm{E})}_{\text{ocean mixing}} + \underbrace{rivPO_4}_{\text{riverine input}} - \underbrace{PP_E}_{\text{primary production}}] \quad \text{(Eq. S21)}$$

$$\frac{d[PO_4^{3-}]_N}{dt}=\frac{1}{Vol_N}\times[\underbrace{(f_{en}+Q_1)\times([PO_4^{3-}]_E-[PO_4^{3-}]_N)+f_{nd}\times([PO_4^{3-}]_D-[PO_4^{3-}]_N)}_{\text{ocean mixing}} - \underbrace{PP_N}_{\text{primary production}}] \quad \text{(Eq. S22)}$$

$$\frac{d[PO_4^{3-}]_I}{dt}=\frac{1}{Vol_I}\times[\underbrace{(f_{si}+Q_3)\times([PO_4^{3-}]_S-[PO_4^{3-}]_I)}_{\text{ocean mixing}}+\underbrace{(f_{id}+Q_2)\times([PO_4^{3-}]_D-[PO_4^{3-}]_I)}_{\text{ocean mixing}} +\underbrace{f_{ib}\times([PO_4^{3-}]_B-[PO_4^{3-}]_I)}_{\text{ocean mixing}}+\underbrace{g\times(1-rom)\times PP_S}_{\text{remineralization}}] \quad \text{(Eq. S23)}$$

$$\frac{d[PO_4^{3-}]_D}{dt}=\frac{1}{Vol_D}\times[\underbrace{f_{ed}\times([PO_4^{3-}]_E-[PO_4^{3-}]_D)}_{\text{ocean mixing}}+\underbrace{(f_{nd}+Q_1)\times([PO_4^{3-}]_N-[PO_4^{3-}]_D)}_{\text{ocean mixing}} +\underbrace{f_{id}\times([PO_4^{3-}]_I-[PO_4^{3-}]_D)}_{\text{ocean mixing}}+\underbrace{(f_{db}+Q_2+Q_3)\times([PO_4^{3-}]_B-[PO_4^{3-}]_D)}_{\text{ocean mixing}} +\underbrace{g\times(1-rom)\times(PP_E+PP_N)}_{\text{remineralization}}] \quad \text{(Eq. S24)}$$

$$\frac{d[PO_4^{3-}]_B}{dt}=\frac{1}{Vol_B}\times[\underbrace{f_{db}\times([PO_4^{3-}]_D-[PO_4^{3-}]_B)}_{\text{ocean mixing}}+\underbrace{(f_{ib}+Q_2+Q_3)\times([PO_4^{3-}]_I-[PO_4^{3-}]_B)}_{\text{ocean mixing}} +\underbrace{(1-g)\times(1-rom)\times(PP_E+PP_N+PP_S)}_{\text{remineralization}}] \quad \text{(Eq. S25)}$$

where $[DIC]_i$, $[ALK]_i$, and $[PO_4^{3-}]_i$ denote the concentrations of DIC, ALK, and PO_4^{3-} in box_i, respectively. Vol_i is the volume of each box. $r_{Corg:P}$ represents the Redfield C:P ratio of organic matter, and the *rain ratio* defines the proportion of PIC to POC exported from the surface ocean. $r_{N:P}$ indicates the Redfield N:P ratio.

2.7 Carbon isotopes

In the numerical implementation, the prognostic tracer for carbon isotopes is the ^{13}C content, denoted C^{13} (mol ^{13}C per unit volume), in each model box. The $\delta^{13}C$ values reported in this study are diagnosed from C^{13} and [DIC] as

$$\delta^{13}C=[\frac{C^{13}/[DIC]}{R_{std}}-1]\times 1000\ (‰),$$

where R_{std} is the standard $^{13}C/^{12}C$ ratio. For any box x, the corresponding isotopic ratio is

$$R_x=R_{std}(1+\frac{\delta^{13}C_x}{1000}),$$

and for the atmosphere,

$$R_A = R_{std}\left(1+\frac{\delta^{13}C_A}{1000}\right).$$

The model prognostically integrates the ^{13}C mass balance for each box, while $\delta^{13}C$ is a diagnostic variable derived from the evolving C^{13} and [DIC] fields at each time step.

The governing equations for C^{13} in the ocean and atmosphere boxes are given as follows:

$$\frac{dC^{13}_S}{dt} = \frac{1}{Vol_S} \times \{ \underbrace{(f_{se}+Q_3)\times(C^{13}_E - C^{13}_S)+\cdots}_{\text{ocean mixing}}$$

$$\underbrace{-\,r_{Corg:p}\times PP_S \times \frac{C^{13}_S}{[DIC]_S}\times\left(1+\frac{\varepsilon_P}{1000}\right)}_{\text{POC production}} \underbrace{-\,rain\ ratio \times r_{Corg:p}\times PP_S\times\frac{C^{13}_S}{[DIC]_S}}_{\text{PIC production}} \quad \text{(Eq. S26)}$$

$$\underbrace{+ff_{dp}\times sol_S \times [pCO_{2,A}\times(R_A\times(1+\frac{\varepsilon_{as}}{1000})) - pCO_{2,S}\times(R_S\times(1+\frac{\varepsilon_{sa}}{1000}))]}_{\text{air-sea exchange}}\}$$

$$\frac{dC^{13}_E}{dt} = \frac{1}{Vol_E} \times \{ \underbrace{f_{se}\times(C^{13}_S - C^{13}_E)+\cdots}_{\text{ocean mixing}}$$

$$\underbrace{-\,r_{Corg:p}\times PP_E \times \frac{C^{13}_E}{[DIC]_E}\times\left(1+\frac{\varepsilon_P}{1000}\right)}_{\text{POC production}} \underbrace{-\,rain\ ratio \times r_{Corg:p}\times PP_E\times\frac{C^{13}_E}{[DIC]_E}}_{\text{PIC production}}$$

$$\underbrace{+ff_{dp}\times sol_E \times [pCO_{2,A}\times(R_A\times(1+\frac{\varepsilon_{as}}{1000})) - pCO_{2,E}\times(R_E\times(1+\frac{\varepsilon_{sa}}{1000}))]}_{\text{air-sea exchange}} \quad \text{(Eq. S27)}$$

$$\underbrace{+\,ORG_{dep}\times[0.995 - \frac{C^{13}_E}{[DIC]_E}\times(1+\frac{\varepsilon_P}{1000})]}_{\text{additional land-derived DIC input and associated shallow-water organic-carbon burial}}$$

$$-\quad \underbrace{Carb_{sh}\times\frac{C^{13}_E}{[DIC]_E}}_{\text{shallow-water carbonate burial}} \quad \underbrace{+\,W_{car}\times(1+\frac{\delta^{13}C_{river}}{1000})}_{\text{riverine input}}\}$$

$$\frac{dC^{13}_N}{dt} = \frac{1}{Vol_N} \times \{ \underbrace{(f_{en}+Q_1)\times(C^{13}_E - C^{13}_N)+\cdots}_{\text{ocean mixing}}$$

$$\underbrace{-\,r_{Corg:p}\times PP_N \times \frac{C^{13}_N}{[DIC]_N}\times\left(1+\frac{\varepsilon_P}{1000}\right)}_{\text{POC production}} \underbrace{-\,rain\ ratio \times r_{Corg:p}\times PP_N\times\frac{C^{13}_N}{[DIC]_N}}_{\text{PIC production}} \quad \text{(Eq. S28)}$$

$$\underbrace{+ff_{dp}\times sol_N \times [pCO_{2,A}\times(R_A\times(1+\frac{\varepsilon_{as}}{1000})) - pCO_{2,N}\times(R_N\times(1+\frac{\varepsilon_{sa}}{1000}))]}_{\text{air-sea exchange}}\}$$

$$\frac{dC_I^{13}}{dt}=\frac{1}{Vol_I}\times[\underbrace{(f_{si}+Q_3)\times(C_S^{13}\text{-}C_I^{13})+\cdots}_{\text{ocean mixing}}$$

$$+\underbrace{g\times(1\text{-}rom)\times r_{Corg:p}\times PP_S\times\frac{C_S^{13}}{[\mathrm{DIC}]_S}\times(1+\frac{\varepsilon_P}{1000})}_{\text{POC remineralization}} \quad \text{(Eq. S29)}$$

$$+\underbrace{g\times rain\ ratio\times r_{Corg:p}\times PP_S\times\frac{C_S^{13}}{[\mathrm{DIC}]_S}}_{\text{PIC dissolution}}]$$

$$\frac{dC_D^{13}}{dt}=\frac{1}{Vol_D}\times[\underbrace{f_{ed}\times(C_E^{13}\text{-}C_D^{13})+\cdots}_{\text{ocean mixing}}$$

$$+\underbrace{g\times(1\text{-}rom)\times r_{Corg:p}\times(PP_E\times\frac{C_E^{13}}{[\mathrm{DIC}]_E}+PP_N\times\frac{C_N^{13}}{[\mathrm{DIC}]_N})\times(1+\frac{\varepsilon_P}{1000})}_{\text{POC remineralization}} \quad \text{(Eq. S30)}$$

$$+\underbrace{g\times rain\ ratio\times r_{Corg:p}\times(PP_E\times\frac{C_E^{13}}{[\mathrm{DIC}]_E}+PP_N\times\frac{C_N^{13}}{[\mathrm{DIC}]_N})}_{\text{PIC dissolution}}]$$

$$\frac{dC_B^{13}}{dt}=\frac{1}{Vol_B}\times[\underbrace{f_{db}\times(C_D^{13}\text{-}C_B^{13})+\cdots}_{\text{ocean mixing}}$$

$$+\underbrace{\begin{matrix}(1\text{-}g)\times(1\text{-}rom)\times r_{Corg:p}\times(PP_E\times\frac{C_E^{13}}{[\mathrm{DIC}]_E}\\ +PP_N\times\frac{C_N^{13}}{[\mathrm{DIC}]_N}+PP_S\times\frac{C_S^{13}}{[\mathrm{DIC}]_S})\times(1+\frac{\varepsilon_P}{1000})\end{matrix}}_{\text{POC remineralization}} \quad \text{(Eq. S31)}$$

$$+\underbrace{(1\text{-}g)\times rain\ ratio\times r_{Corg:p}\times(PP_E\times\frac{C_E^{13}}{[\mathrm{DIC}]_E}+PP_N\times\frac{C_N^{13}}{[\mathrm{DIC}]_N}+PP_S\times\frac{C_S^{13}}{[\mathrm{DIC}]_S})}_{\text{PIC dissolution}}$$

$$\underbrace{-\,Carb_{bottom}\times(PP_E\times\frac{C_E^{13}}{[\mathrm{DIC}]_E}+PP_N\times\frac{C_N^{13}}{[\mathrm{DIC}]_N}+PP_S\times\frac{C_S^{13}}{[\mathrm{DIC}]_S})/PP_{E+N+S}}_{\text{bottom-water carbonate burial}}]$$

$$\frac{dP^{13}_{\mathrm{CO_{2},A}}}{dt}=\frac{1}{V_{atm}}\times\{\underbrace{-ff_{dp}\times[sol_{S}\times(p\mathrm{CO_{2,A}}\times(R_{A}\times(1+\frac{\varepsilon_{as}}{1000}))-p\mathrm{CO_{2,S}}\times(R_{S}\times(1+\frac{\varepsilon_{sa}}{1000})))}_{\text{air-sea exchange}}$$

$$+\underbrace{sol_{E}\times(p\mathrm{CO_{2,A}}\times(R_{A}\times(1+\frac{\varepsilon_{as}}{1000}))-p\mathrm{CO_{2,E}}\times(R_{E}\times(1+\frac{\varepsilon_{sa}}{1000})))}_{\text{air-sea exchange}}$$

$$+\underbrace{sol_{N}\times(p\mathrm{CO_{2,A}}\times(R_{A}\times(1+\frac{\varepsilon_{as}}{1000}))-p\mathrm{CO_{2,N}}\times(R_{N}\times(1+\frac{\varepsilon_{sa}}{1000})))]}_{\text{air-sea exchange}} \quad \text{(Eq. S32)}$$

$$+\underbrace{DG_{volkero}\times(1+\frac{\delta^{13}\mathrm{C_{volkero}}}{1000})}_{\text{volcanic degassing and kerogen oxidation}}\ \}$$

Each ^{13}C flux is obtained by multiplying the corresponding DIC flux by the isotopic ratio of its source reservoir. Internal ocean fluxes adopt the C^{13}/[DIC] ratio of the donor box. Riverine fluxes are assigned an isotopic ratio of $(1+\delta^{13}C_{river}/1000)$, and air-sea exchange terms use R_A and the surface-ocean ratio R_x together with temperature-dependent fractionation factors. Hence, the structure of the ^{13}C equations parallels that of the DIC equations, with additional terms explicitly accounting for isotopic fractionation.

In the equatorial surface box “E”, Eq. S27 includes an additional term:

$$ORG_{dep} \times [0.995 - (C^{13}_{E} / [DIC]_{E})\ (1 + \varepsilon_P / 1000)]$$

which represents the shallow-water organic-carbon burial flux. This term is defined as the additional land-derived inorganic-carbon input that balances the surface-ocean carbon budget. In this formulation, the component $0.995 \times ORG_{dep}$ corresponds to an external inorganic carbon input carrying $\delta^{13}C$=-5‰, whereas the component $-ORG_{dep} \times (C^{13}_{E} / [DIC]_{E}) \times (1 + \varepsilon_P / 1000)$ represents its burial as organic carbon after photosynthetic fractionation. These two components offset each other in the DIC budget, leaving the total amount of DIC unchanged while modifying the isotopic composition of the surface ocean. The resulting $\delta^{13}C$ signals established in the surface boxes are subsequently transmitted to deeper layers through the remineralization and dissolution of particulate organic and inorganic carbon.

For all air-sea exchange terms, ff_{dp} (set to 0.9995) represents the air-sea CO_2 disequilibrium factor, and ε_p denotes the isotopic fractionation associated with algal POC production. PIC is assumed to be in isotopic equilibrium with ambient seawater, so carbonate shell formation introduces negligible additional fractionation beyond that implied by the local C^{13} / [DIC] ratio.

The isotopic fractionation associated with air-sea CO_2 exchange is temperature-dependent and is represented in the model by the following expressions(*104*):

$$\varepsilon_{as}=0.19-\frac{373}{\mathrm{T}}\text{‰} \quad \text{(Eq. S33)}$$

$$\varepsilon_{sa}=\frac{\left(24.12-\frac{9866}{T}\right)[HCO_3^-]-7.1\times[CO_3^{2-}]}{[CO_2]+[HCO_3^-]+[CO_3^{2-}]} \text{ (Eq. S34).}$$

3. Experimental design

To investigate the sensitivity of the carbon cycle to orbital forcing and to disentangle the relative contributions of individual processes, we performed a series of model simulations comprising one fully integrated experiment and four targeted sensitivity experiments. All model results are provided in the printout files included in the uploaded Code S1 package.

The model is first initialized from a steady-state configuration and integrated for 2 Myr to ensure that the system reaches equilibrium (Table S4). Following the spin-up phase, the model is forced by orbital variations, represented by the combined ETP signal(*6*), and extended for an additional 4 Myr.

3.1 Integrated experiment

The fully coupled run serves as the reference simulation, where the baseline fluxes of carbonate and silicate weathering (f_{car} and f_{sil}), riverine PO_4^{3-} input ($rivPO_4$), and shallow-water carbonate burial ($Carb_{sh}$) are modulated by orbital variations. Specifically, f_{car} = 10.7 × 10^{12} × (1 + 0.3 × ETP) mol yr^{-1}, f_{sil} = 5.0 × 10^{12} × (1 + 0.15 × ETP) mol yr^{-1}, $rivPO_4$ = W_{sil} / 200, and $Carb_{sh}$ = 9 × 10^{12} × (1 + ETP) mol yr^{-1}.

This configuration captures the integrated response of the carbon cycle to orbitally driven changes in weathering inputs, riverine nutrient supply, and carbonate burial dynamics.

3.2 Sensitivity experiments

To isolate the effects of individual processes, we conducted four additional experiments with stepwise modifications relative to the baseline configuration:

Experiment 1: Constant PO_4^{3-} supply. This experiment is identical to the integrated simulation except that the riverine PO_4^{3-} flux is held constant at $rivPO_4$ = 2.5 × 10^{10} mol yr^{-1}, thereby suppressing the orbital modulation of nutrient delivery.

Experiment 2: Constant PO_4^{3-} input and shallow-water $CaCO_3$ burial. Building on Experiment 1, shallow-water $CaCO_3$ burial is prescribed as a constant value ($Carb_{sh}$ = 9 × 10^{12} mol yr^{-1}), eliminating variability in both nutrient delivery and shelf carbonate burial.

Experiment 3: Constant weathering fluxes. In this configuration, the baseline fluxes of carbonate and silicate weathering are kept fixed (f_{car} = 10.7 × 10^{12} mol yr^{-1}, f_{sil} = 5.0 × 10^{12} mol yr^{-1}), while PO_4^{3-} input remains orbitally paced ($rivPO_4$ = 5.0 × 10^{10} × (1 + 0.15 × ETP) mol yr^{-1}), and shallow-water $CaCO_3$ burial continues to vary in response to orbital forcing.

Experiment 4: Constant weathering fluxes and shallow-water $CaCO_3$ burial. Following the setup of Experiment 3, shallow-water $CaCO_3$ burial is also prescribed as a constant, thereby removing orbital variability from both weathering and burial terms.

We parameterize shallow-water $CaCO_3$ burial as a function of ETP, representing a process-based simplification that links shelf carbonate deposition to orbital forcing through its primary environmental controls. On orbital timescales, eccentricity-paced sea-level fluctuations regulate accommodation space on continental shelves, periodically expanding or contracting the areas suitable for carbonate accumulation. At the same time, orbitally

driven variations in hydrological intensity and chemical weathering modulate riverine DIC and ALK fluxes, thereby influencing carbonate saturation, production, and biocalcification rates in shallow-marine settings. By scaling burial to ETP, our model captures the integrated response of shelf carbonate systems to these coupled processes.

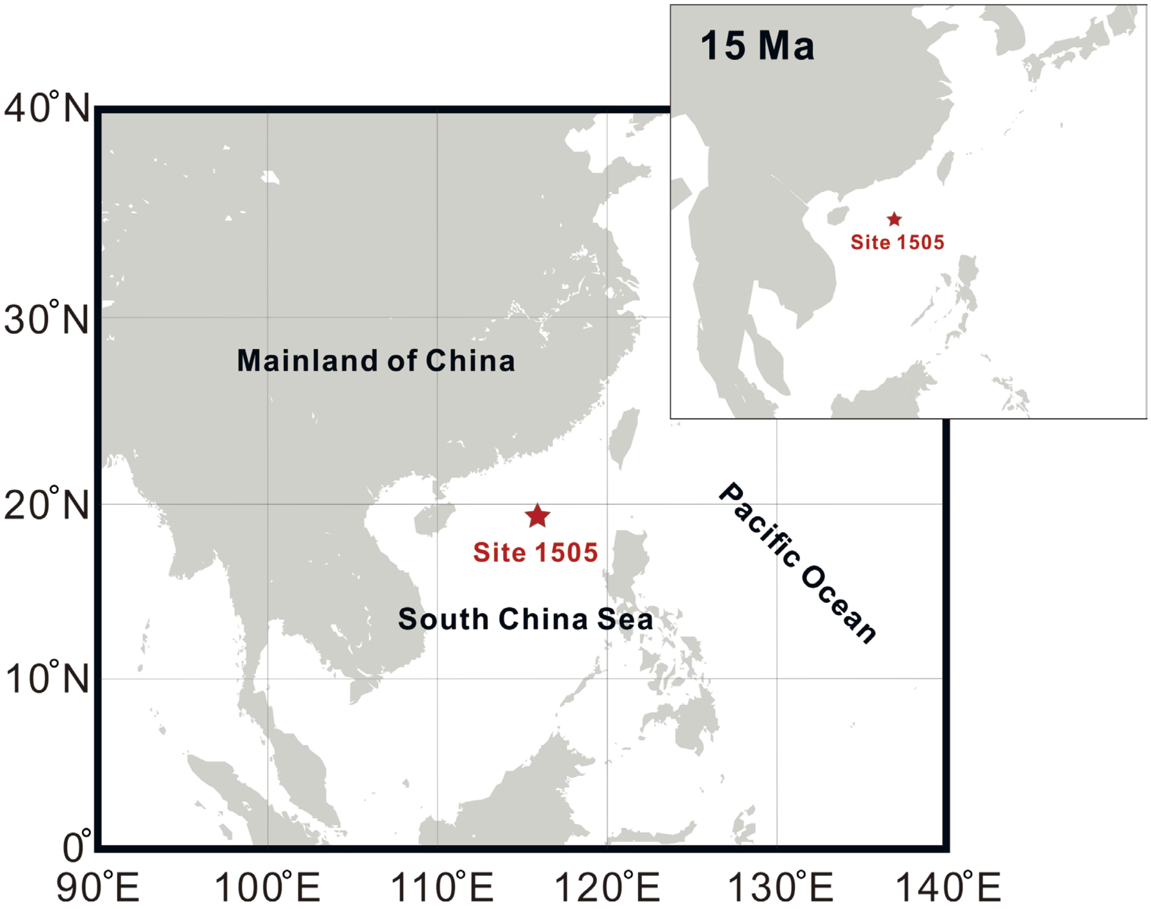

Fig. S1 Location of IODP Site U1505 in the northern South China Sea(*17*)**.** The upper-right inset presents a paleogeographic reconstruction at 15 Ma(*18*).

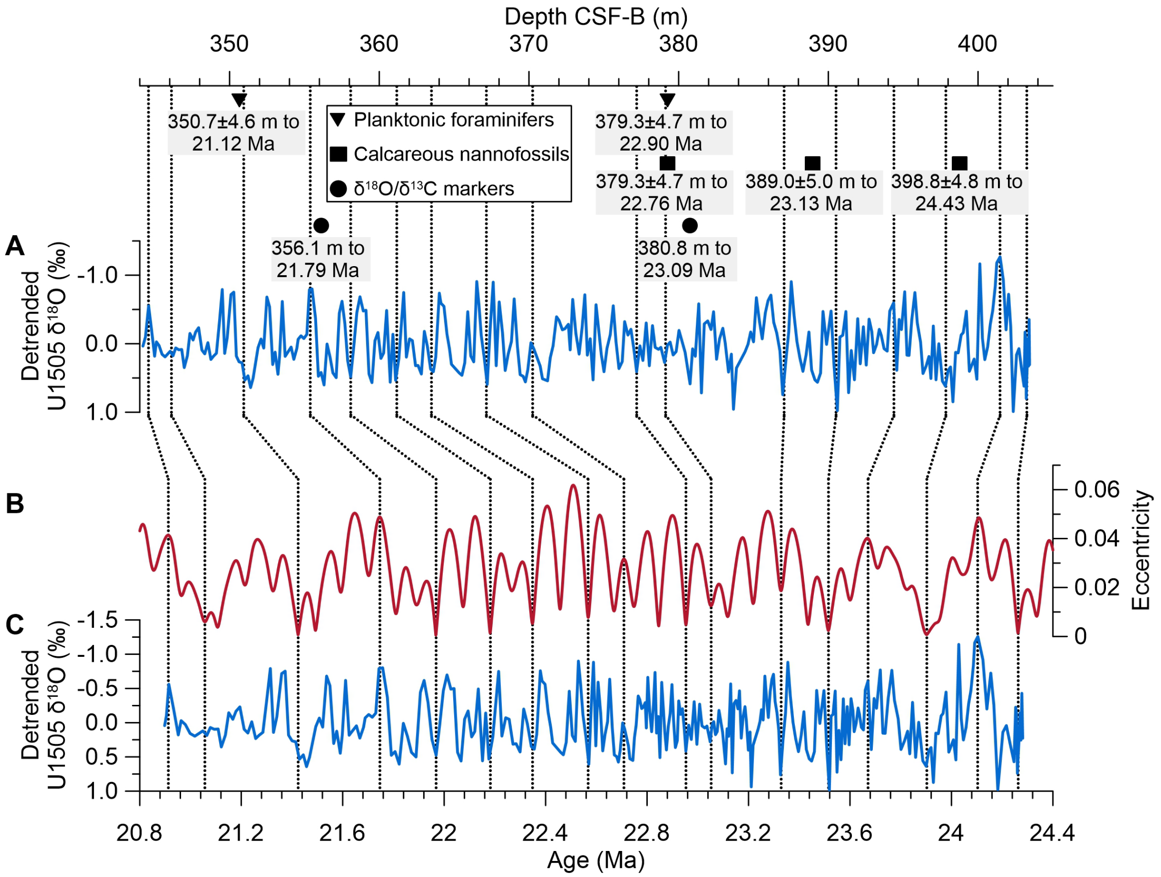

Fig. S2 Astronomically tuned age model for Site U1505 covering 24.3-21 Ma, derived from the correlation of benthic foraminiferal $\delta^{18}O$ with eccentricity. (A) Detrended benthic $\delta^{18}O$ versus core depth. The triangles, squares, and circles represent planktonic foraminifera datums(*17*), calcareous nannofossil datums(*17*), and isotope markers, respectively. **(B)** Eccentricity(*6*). **(C)** Detrended benthic $\delta^{18}O$ versus age. Grey dashed lines show their correlations.

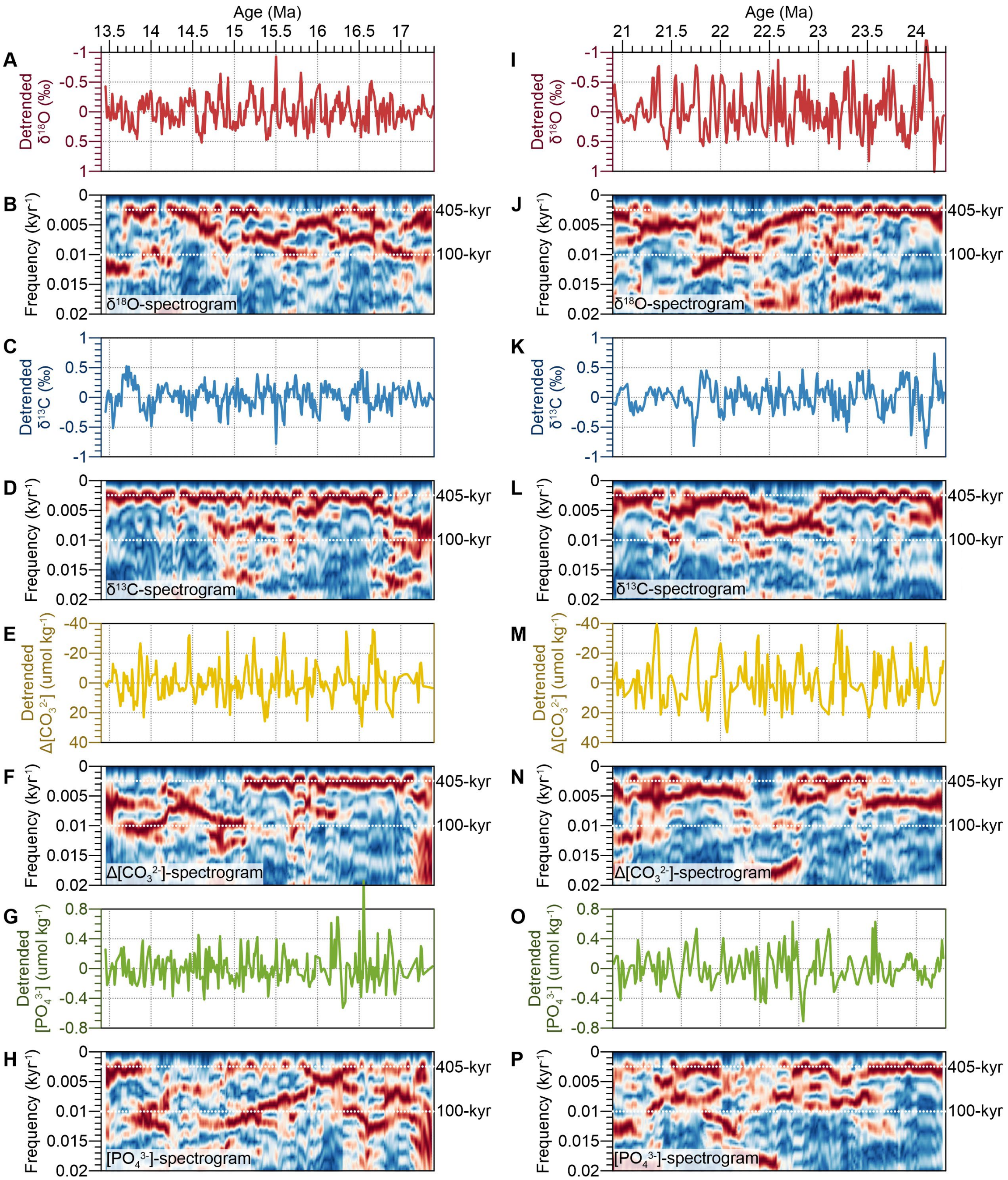

Fig. S3 Time-frequency analysis results for Site U1505. (A, B, I, J) Detrended benthic $\delta^{18}O$, **(C, D, K, L)** $\delta^{13}C$, **(E, F, M, N)** $\Delta[CO_3^{2-}]$, and **(G, H, O, P)** $[PO_4^{3-}]$**,** along with their corresponding evolutionary multi-taper spectrograms calculated using a 500-kyr window.

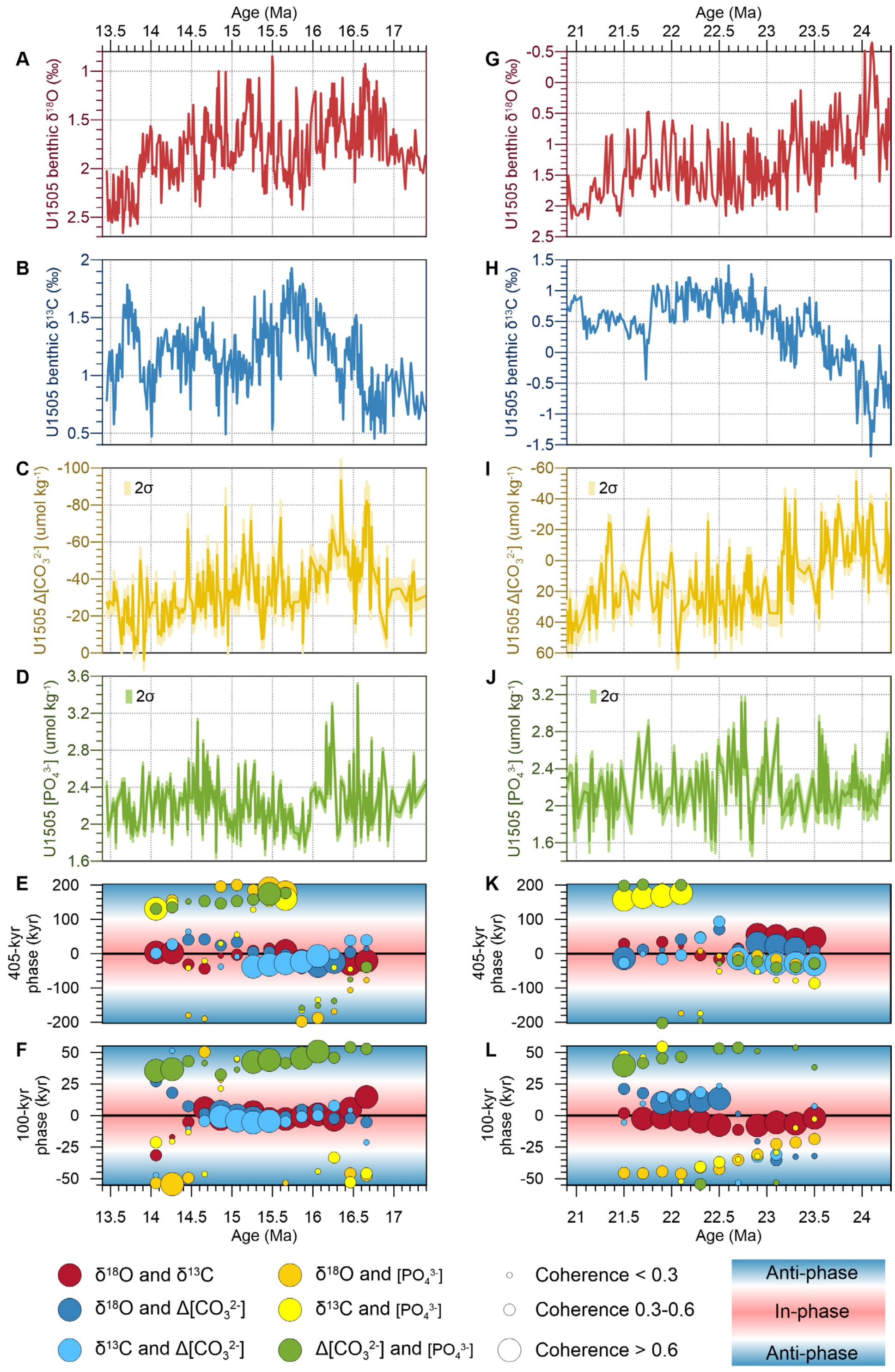

Fig. S4 Temporal trends of proxies at Site U1505. (A, G) Benthic foraminiferal $\delta^{18}O$, **(B, H)** $\delta^{13}C$, **(C, I)** $\Delta[CO_3^{2-}]$, and **(D, J)** $[PO_4^{3-}]$ records from Site U1505. **(E, F, K, L)** Evolutionary phase relationships of $\delta^{18}O$-$\delta^{13}C$, $\delta^{18}O$-$\Delta[CO_3^{2-}]$, $\delta^{13}C$-$\Delta[CO_3^{2-}]$, $\delta^{18}O$-$[PO_4^{3-}]$, $\delta^{13}C$-$[PO_4^{3-}]$, and $\Delta[CO_3^{2-}]$-$[PO_4^{3-}]$ on eccentricity timescales. These plots illustrate the anti-phase behavior between $[PO_4^{3-}]$ and $\delta^{18}O$/$\delta^{13}C$/$\Delta[CO_3^{2-}]$ at 405- and 100-kyr cycles.

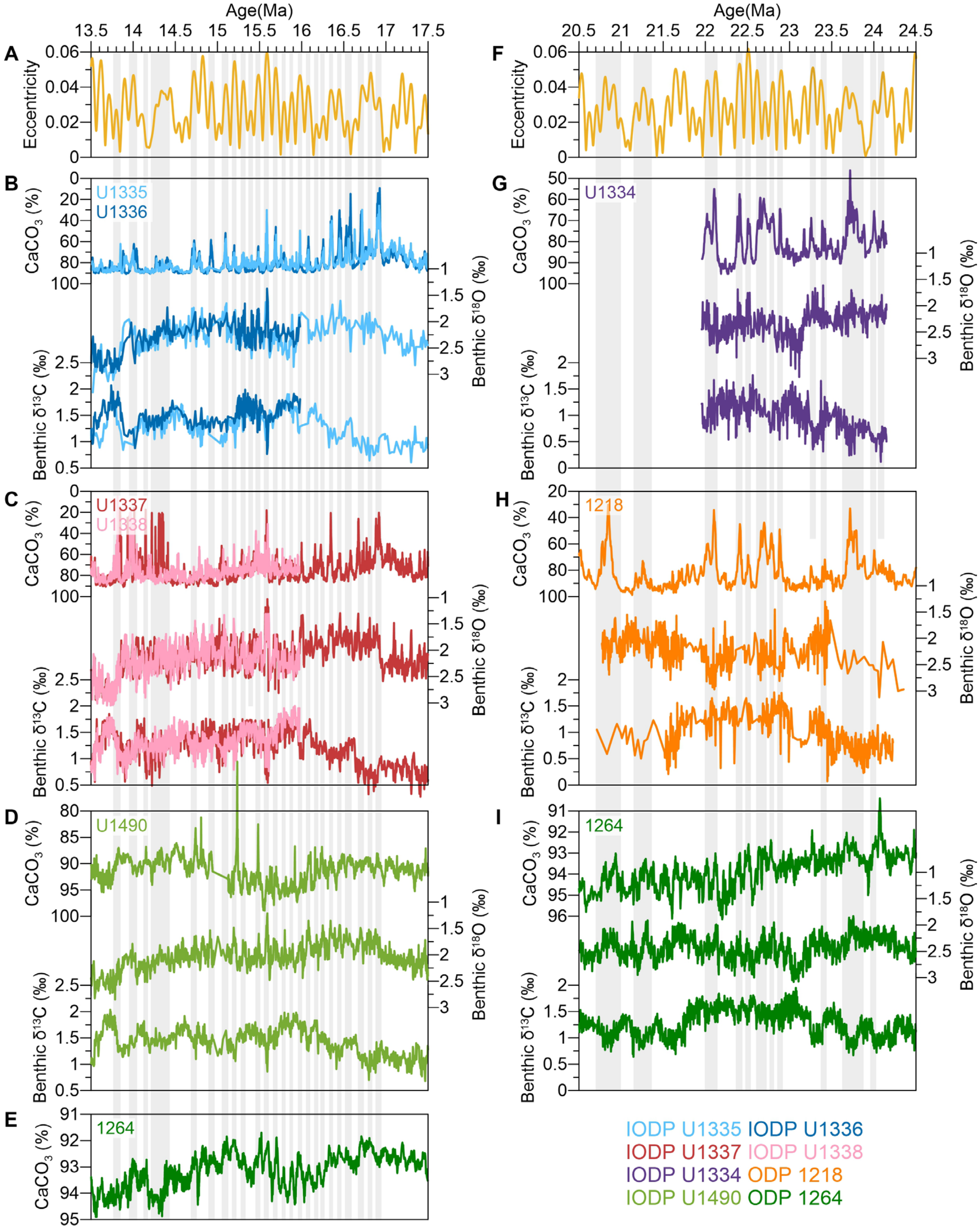

Fig. S5 Comparison of %$CaCO_3$ and stable isotopes on eccentricity timescales. (A, F) Eccentricity(*6*). **(B-D, G, H)** %$CaCO_3$ at Pacific Sites 1218, U1334, U1335, U1336, U1337, U1338, U1490, and **(E, I)** Atlantic Site 1264, accompanied by the corresponding benthic $\delta^{18}O$ and $\delta^{13}C$ data from each site(*5*, *21–27*, *34–39*, *41*).

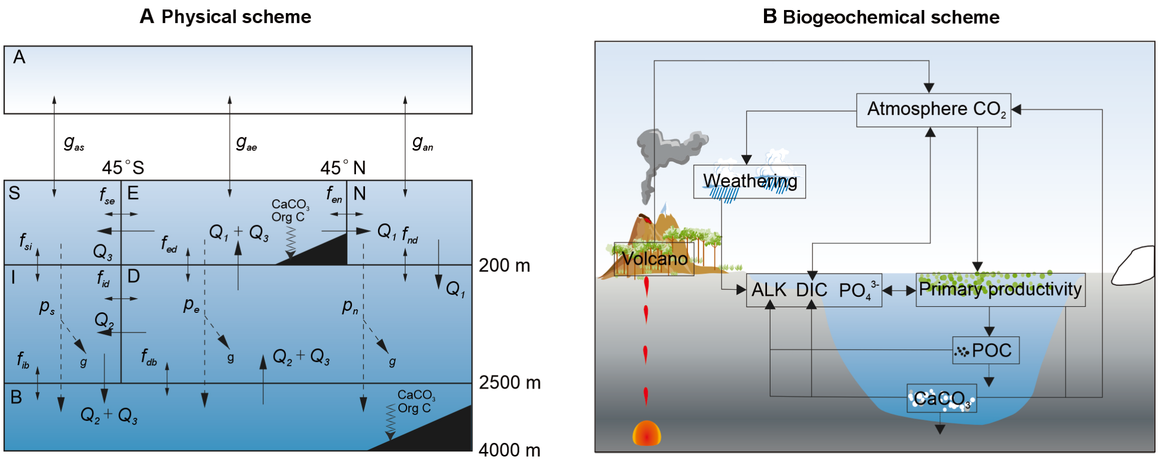

Fig. S6 Model configuration(*9*, *12*)**.** **(A)** Physical scheme. **(B)** Biogeochemical scheme.

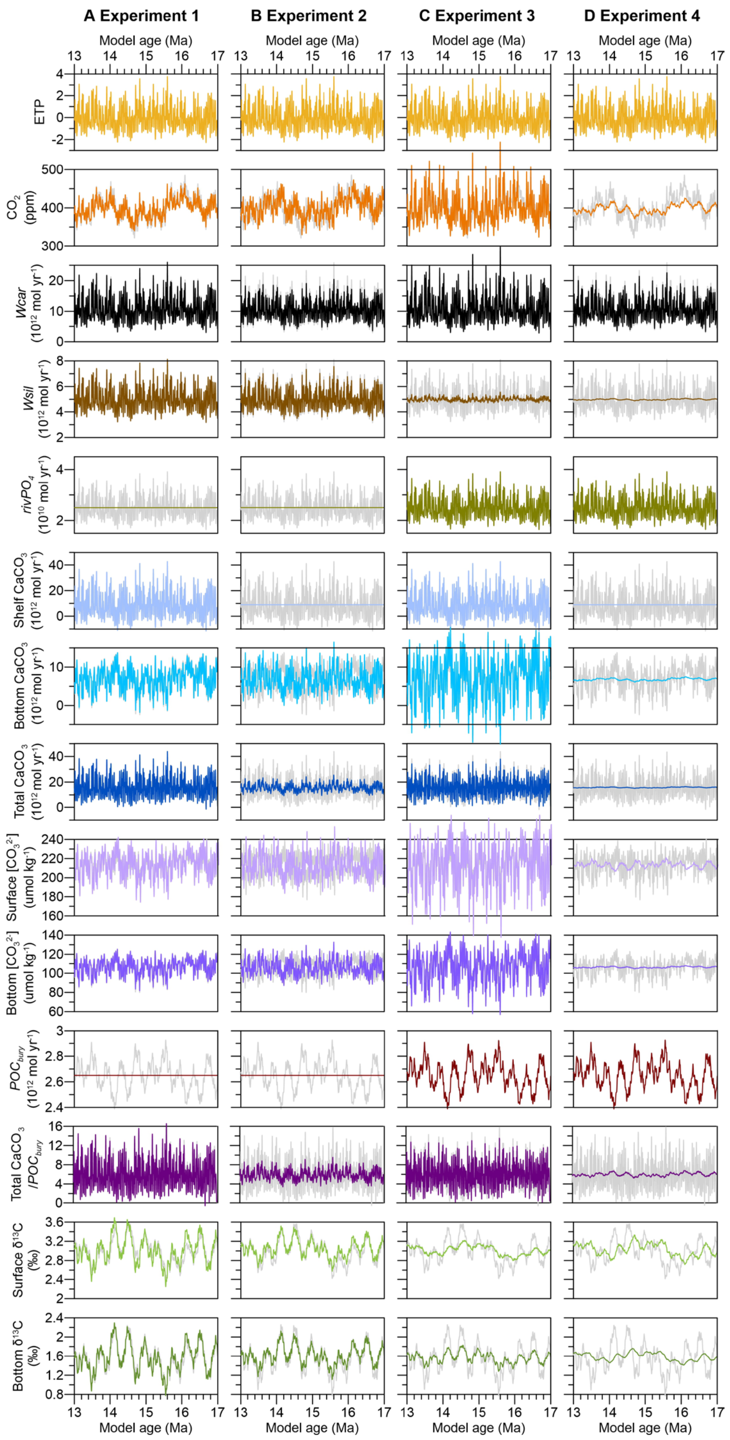

Fig. S7 Simulated fluctuations of the marine carbon cycle on orbital timescales from the four sensitivity experiments. **(A)** Experiment 1: Identical to the integrated Experiment, except that riverine PO_4^{3-} input is prescribed as a constant value. **(B)** Experiment 2: As in Experiment 1, but shallow-water $CaCO_3$ burial is fixed. **(C)** Experiment 3: Baseline fluxes of carbonate and silicate weathering are held constant, while riverine PO_4^{3-} input and shallow-water $CaCO_3$ burial remain orbitally forced. **(D)** Experiment 4: Same as Experiment 3, but with constant shallow-water $CaCO_3$ burial.

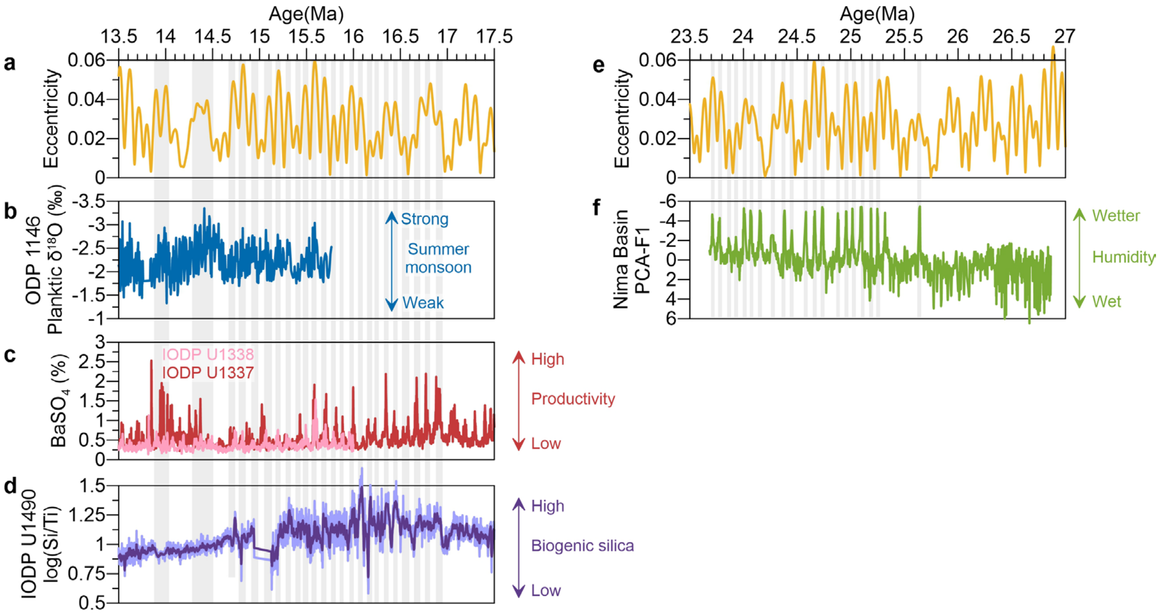

Fig. S8 Proxies from terrestrial and marine regimes depicting the evolution of the hydrological cycle and sea surface productivity in eccentricity bands. (A, E) Eccentricity(*6*). **(B)** Planktic foraminiferal $\delta^{18}O$ from Site 1146, with lower $\delta^{18}O$ values signifying relatively strong monsoon intensity during eccentricity maxima(*43*). **(C)** Variations in $BaSO_4$ content (%) at Sites U1337 and U1338 reflect changes in export production(*25–27*). **(D)** log(Si/Ti) from Site U1490 as an indicator of biogenic silicate (opal)(*23*). **(F)** PCA-F1 (the first principal component of Redness, SIRM (saturation isothermal remanent magnetization), S-ratio, and HIRM (hard IRM)) from the Nima Basin, central Tibet, illustrates strong 100-kyr cycles of the local hydroclimate(*44*).

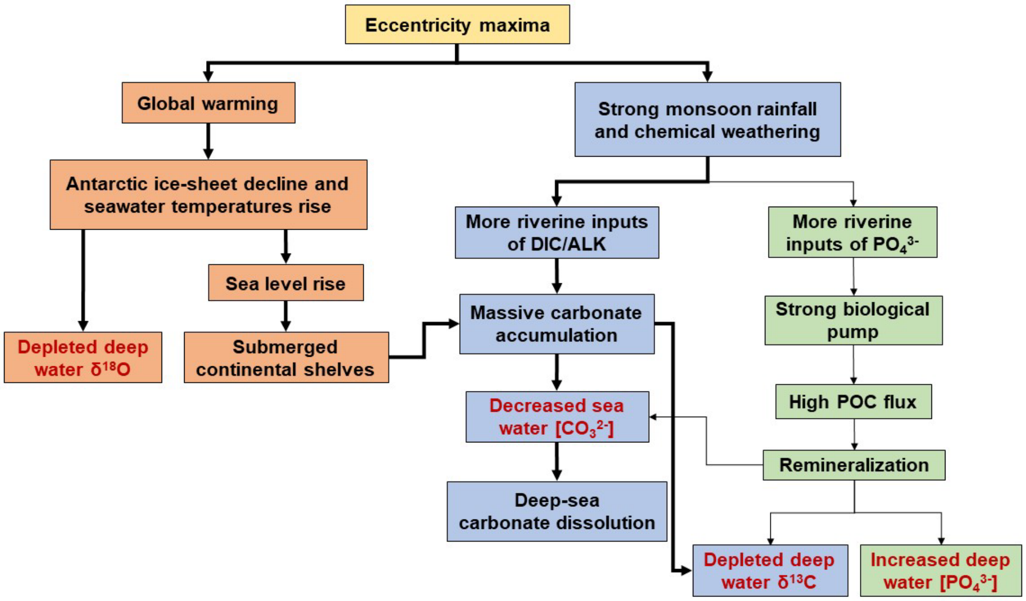

Fig. S9 Schematic diagram illustrating the "chemical weathering and shelf-basin carbonate partitioning" hypothesis during periods of eccentricity maxima.

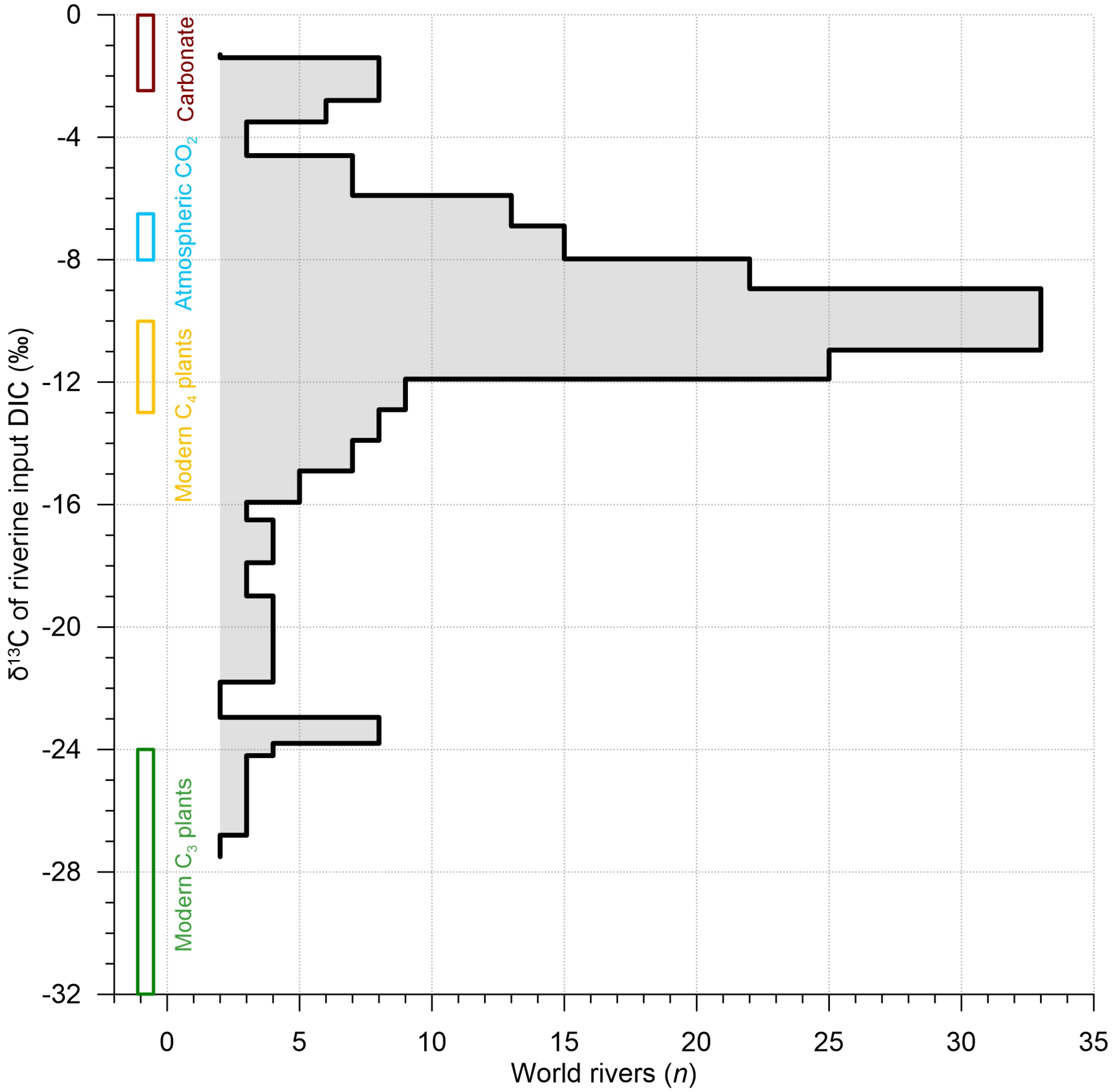

Fig. S10 Compilation of $\delta^{13}C$ values of DIC in global rivers along with the $\delta^{13}C$ signatures of major carbon sources. The $\delta^{13}C$ composition of riverine DIC is primarily determined by the dominant weathering source. DIC derived from carbonate weathering represents a mixture of carbon from carbonate dissolution (-2.5‰ to 0‰) and from the consumption of atmospheric CO_2 (-8‰ to -6.5‰) or soil CO_2 (-32‰ to -24‰ for C_3 plants and -13‰ to -10‰ for C_4 plants), whereas DIC produced by silicate weathering is entirely sourced from atmospheric or soil CO_2(*105–107*). Globally, $\delta^{13}C$-DIC values measured in rivers ($n = 241$) typically range from -12‰ to -5‰(*52*, *53*). Although soil CO_2 constitutes a major source of ^{12}C, carbon released through soil respiration and organic matter decomposition operates on relatively short timescales. Because our model is designed to resolve isotope dynamics on orbital timescales, such "fast-cycle" processes are not considered here, and the analysis instead focuses on long-term geological fluxes. Accordingly, a $\delta^{13}C$ value of -5‰ is adopted as a relatively heavy isotopic endmember for riverine input.

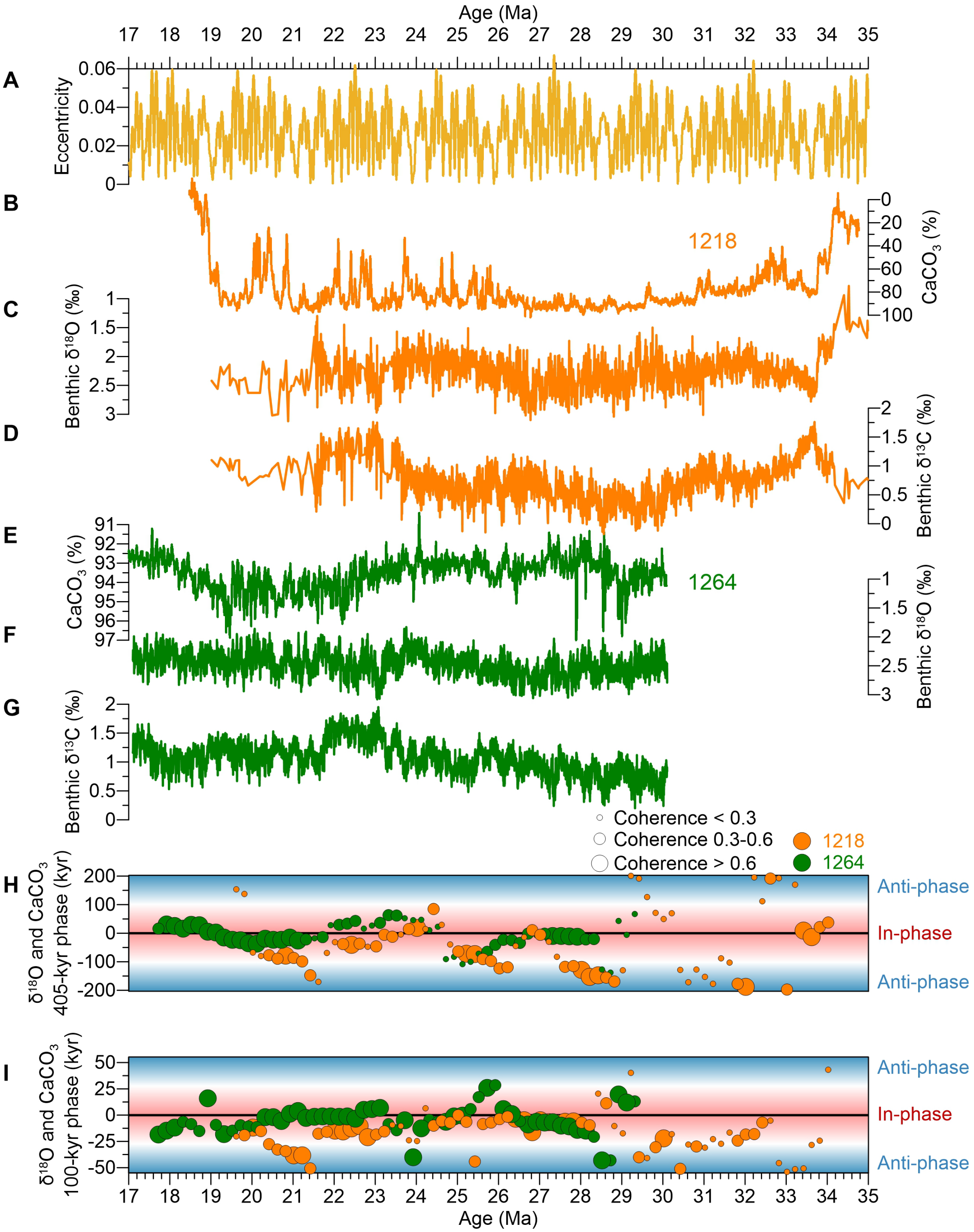

Fig. S11 Eccentricity-scale phase coupling between %$CaCO_3$ and stable isotopes during the Oligocene. (A) Eccentricity(*6*). **(B-D)** %$CaCO_3$ records and corresponding benthic $\delta^{18}O$ and $\delta^{13}C$ data from the Pacific Site 1218(*5*). **(E-G)** Same as (B-D) but for the Atlantic Site 1264(*22*, *24*, *41*). **(H, I)** Evolutionary phase relationships of parallel $\delta^{18}O$ and %$CaCO_3$ time series at the 405- and 100-kyr cycles, both showing an in-phase pattern.

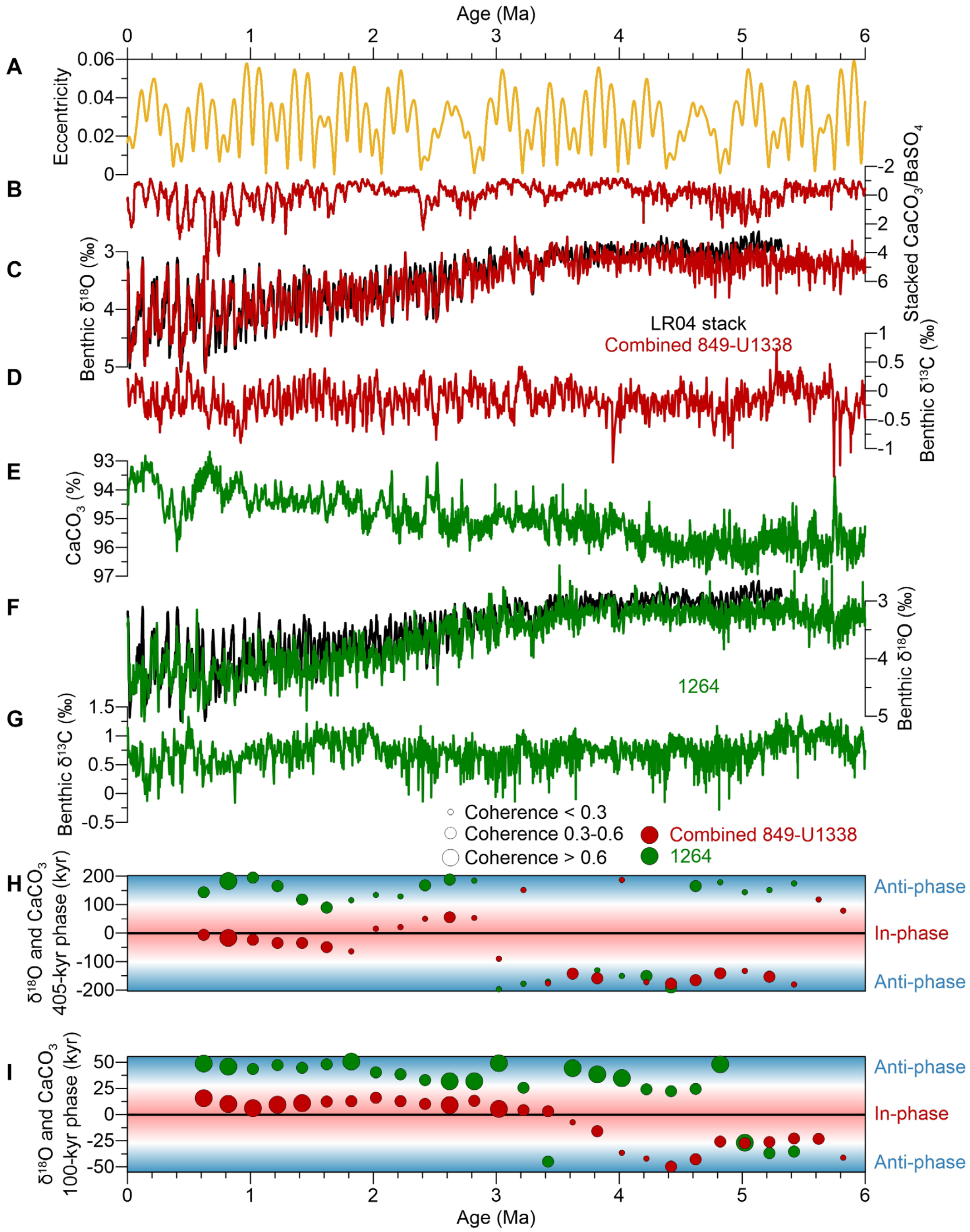

Fig. S12 Eccentricity-scale phase coupling between %$CaCO_3$ and stable isotopes during the Pliocene and Pleistocene. (A) Eccentricity(*6*). **(B-D)** A stacked $CaCO_3/BaSO_4$ record reflecting %$CaCO_3$ dissolution cycles, along with combined benthic $\delta^{18}O$ and $\delta^{13}C$ data from Pacific Sites U1338 and 849(*25*, *27*, *74*, *75*). The black line denotes the LR04 global benthic $\delta^{18}O$ stack(*73*). **(E-G)** %$CaCO_3$ records and corresponding benthic $\delta^{18}O$ and $\delta^{13}C$ data from the Atlantic Site 1264(*22*, *24*, *41*, *68*). **(H, I)** Evolutionary phase relationships between parallel $\delta^{18}O$ and %$CaCO_3$ time series in the 405- and 100-kyr bands, both showing an anti-phase pattern that shifts to in-phase coupling in the composite Pacific record after ~3 Ma.

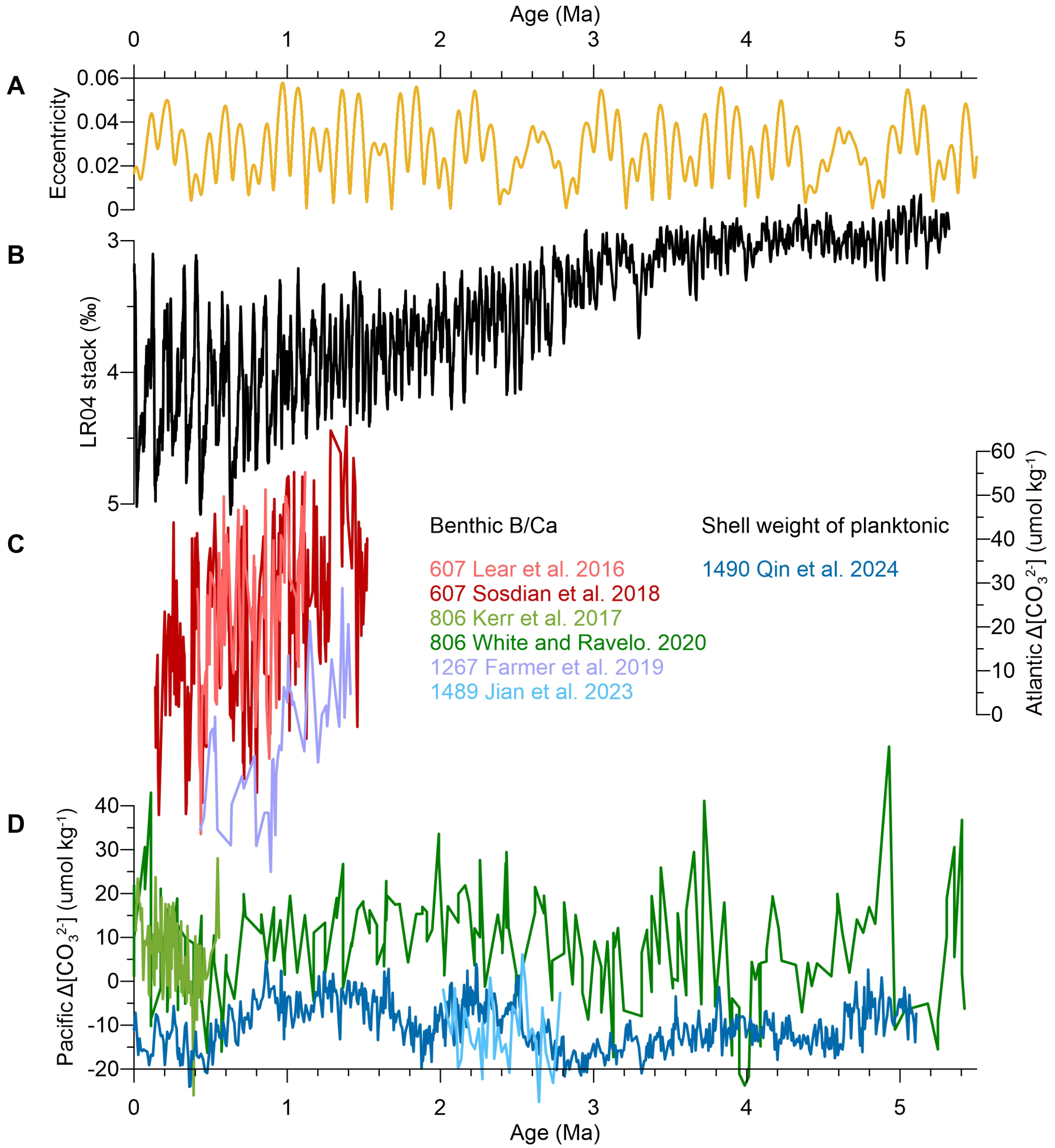

Fig. S13 Compilation of global deep-sea Δ[CO_3^{2-}] records since the Pliocene. (A) Eccentricity(*6*). **(B)** The LR04 global benthic δ^{18}O stack(*73*). **(C)** Atlantic Δ[CO_3^{2-}] records(*69*, *72*, *77*). **(D)** Pacific Δ[CO_3^{2-}] records(*70*, *71*, *76*, *78*). The magnitude, timing, and structure of deep Atlantic Ocean Δ[CO_3^{2-}] variations closely parallel changes in %$CaCO_3$, in contrast to the small-amplitude, anti-phase fluctuations in Δ[CO_3^{2-}] and %$CaCO_3$ observed in the Pacific during the mid-to-late Pleistocene. The B/Ca-to-Δ[CO_3^{2-}] conversion is based on published calibrations(*29*, *83–86*) and accounts only for past variations in seawater [B] when correcting B/Ca values (see Methods). Unlike other records, the IODP Site U1490 reconstruction relies on planktonic foraminiferal shell weights(*76*).

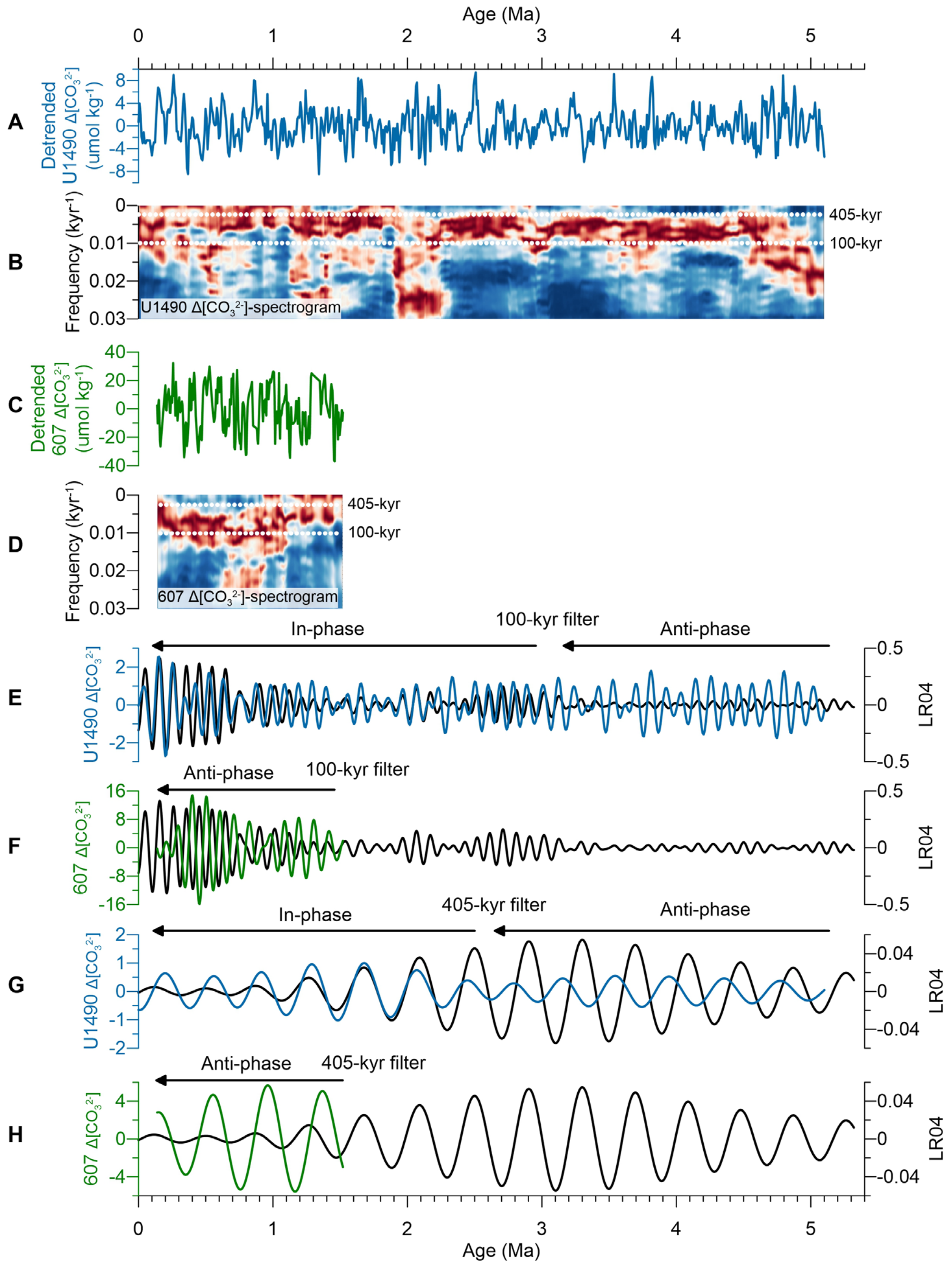

Fig. S14 Eccentricity-scale phase coupling between deep-sea Δ[CO_3^{2-}] and the LR04 stack since the Pliocene. (A, B) Detrended Δ[CO_3^{2-}] records from Site U1490(*76*), and **(C, D)** Site 607(*77*), along with their corresponding evolutionary multi-taper spectrograms calculated using a 500-kyr window. **(E-H)** Bandpass-filtered Δ[CO_3^{2-}] records from Sites U1490 and 607, and the LR04 stack, in the 405- and 100-kyr eccentricity bands. The spectrograms reveal relatively weak power at eccentricity periods, with the 100-kyr component of Δ[CO_3^{2-}] becoming more prominent only within the past ~1 Ma. The filtering results show that Δ[CO_3^{2-}] variations at Site U1490 were anti-phased with the LR04 stack between ~5 and 3 Ma, and in-phase after ~3 Ma. At Site 607, Δ[CO_3^{2-}] variations became anti-phased with the LR04 stack after ~1.4 Ma within the eccentricity bands.

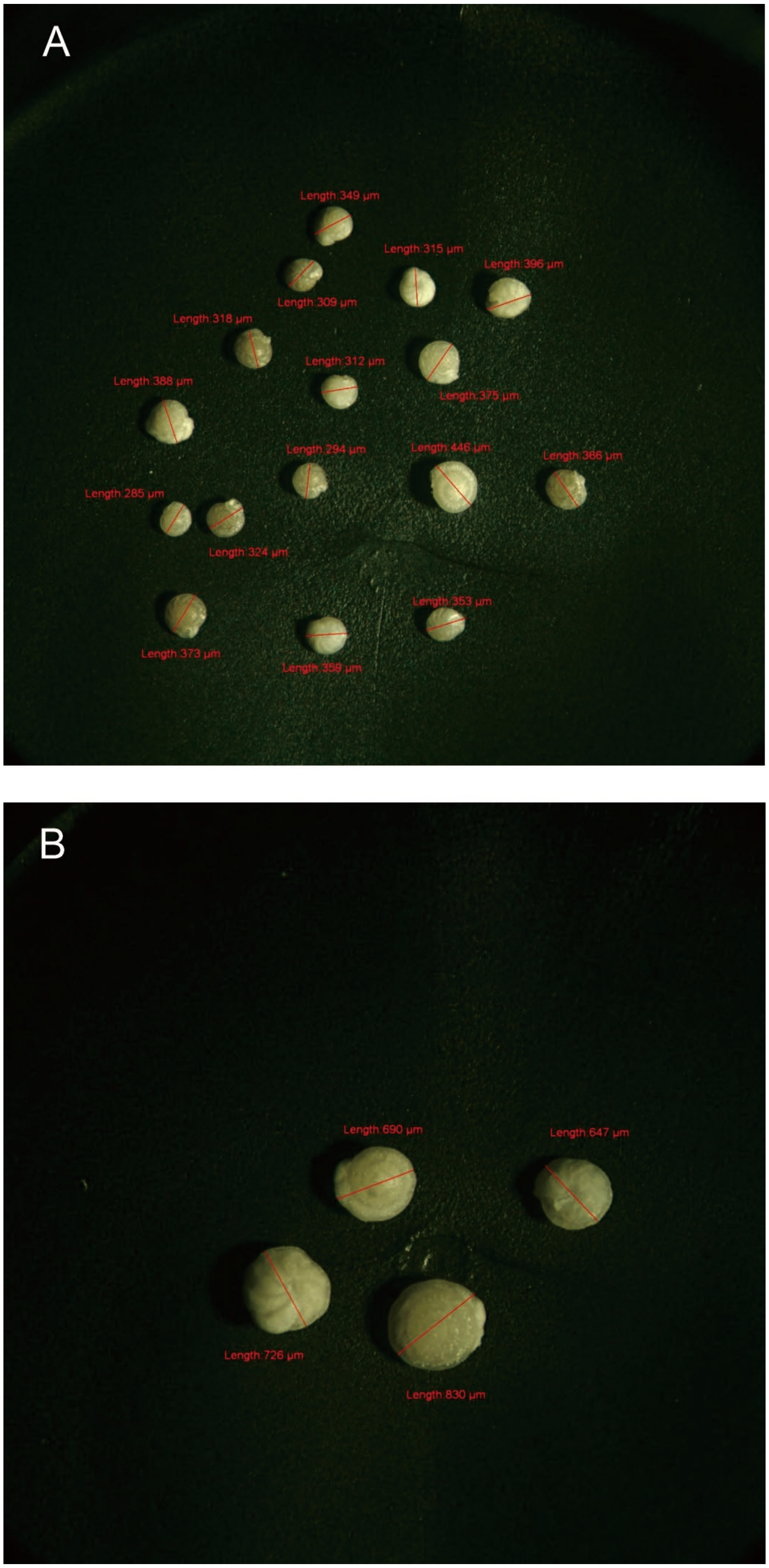

Fig. S15 Specimens of *Cibicidoides mundulus* used for trace element analysis, categorized by shell size: (A) 250-500 μm and **(B)** 500-850 μm.

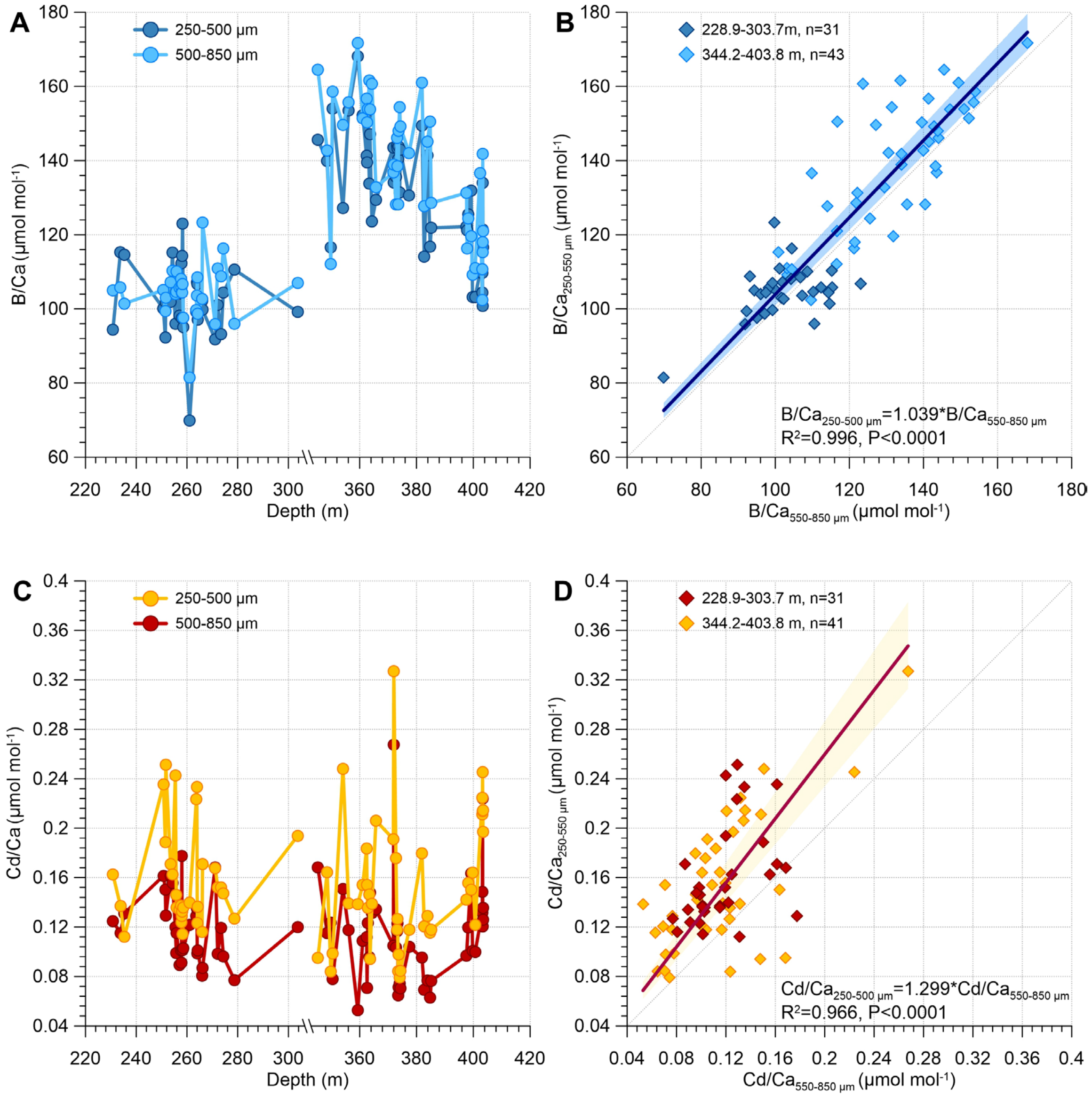

Fig. S16 Correction of B/Ca and Cd/Ca for shell-size effects. (A, C) Comparisons of B/Ca and Cd/Ca values between two shell size ranges, 250-500 μm and 500-850 μm, obtained from identical samples at various depths. **(B, D)** Linear regression of B/Ca and Cd/Ca across the two shell size ranges, 250-500 μm and 500-850 μm.

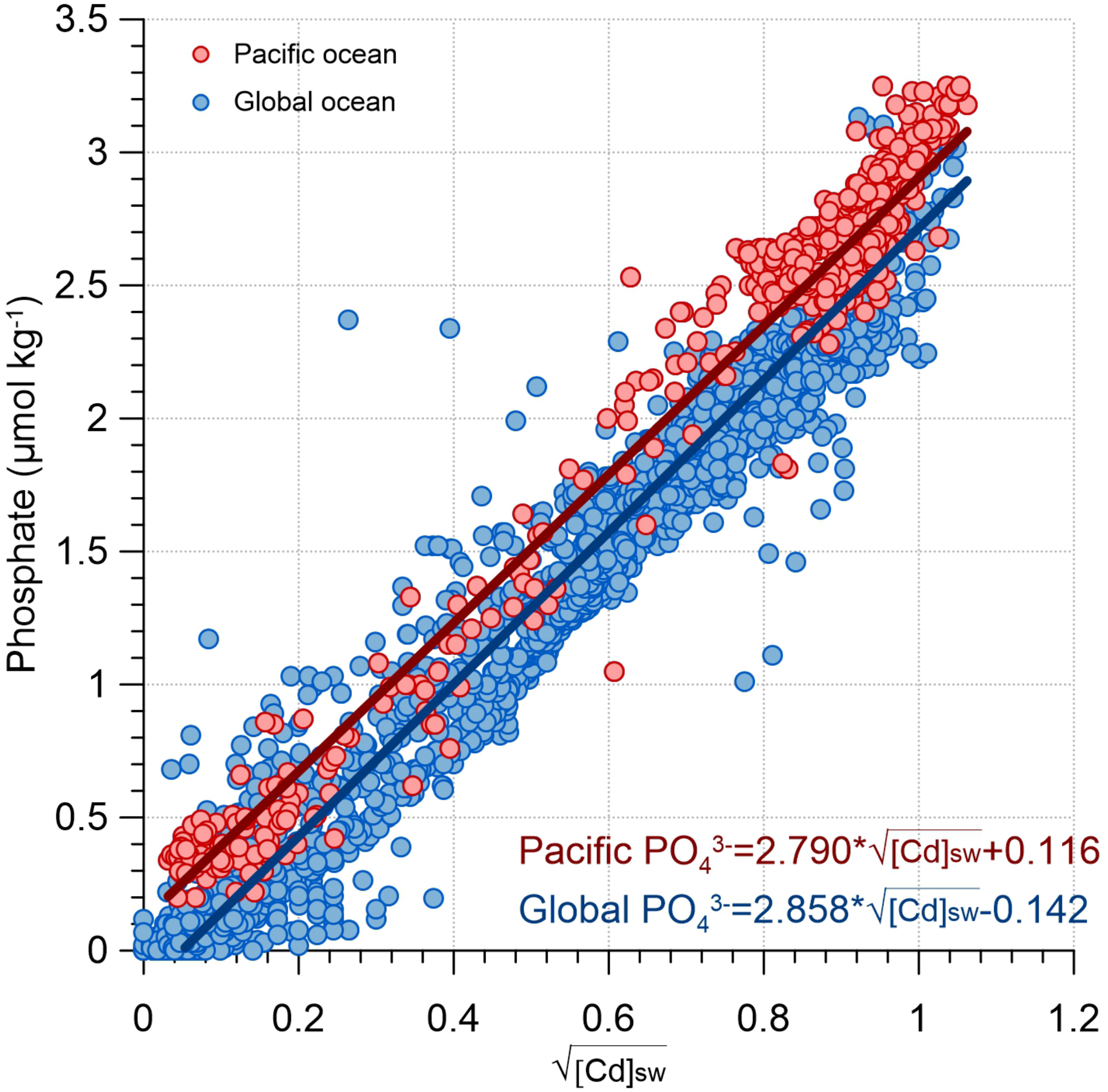

Fig. S17 The relationship between $[PO_4^{3-}]$ and $\sqrt{[Cd]_{sw}}$ at all depths in global oceans, generated from the GEOTRACES 2021 dataset(*96*, *97*)**.** Red dots represent data from the Pacific Ocean, while blue dots indicate global data (including the Pacific Ocean). Solid red and blue lines depict the least squares regression equations between $[PO_4^{3-}]$ and $\sqrt{[Cd]_{sw}}$.

Table S1. Overview of all individual ODP/IODP sites discussed in this study. All sites are shown in (Fig. 3M).

Site	Longitude	Latitude	Region	Water depth (m)	Benthic isotopes source	$\Delta[CO_3^{2-}]$ source	%$CaCO_3$ source	Age model source
IODP U1505	115°51.5370′E	18°55.0570′N	West Pacific	2916.6	(*9*), *This study*	*This study*	-	(*9*),*This study*
IODP U1490	142°39.27′E	05°48.95′N	West Pacific	2341	(*23*)	(*76*)	(*23*)	(*23*, *76*)
IODP U1489	141°1.67′E	2°7.19′N	West Pacific	3421	-	(*70*)	-	(*70*)
ODP 806	159°12′E	0°12′N	West Pacific	2520	-	(*71*, *78*)	-	(*71*, *78*)
IODP U1338	117°58.178′W	2°30.469′N	East Pacific	4200	(*34*, *75*)	-	(*25*, *27*, *74*, *75*)	(*27*, *34*, *36*, *75*)
IODP U1337	123°12.352′W	3°50.009′N	East Pacific	4463	(*35*, *38*, *75*)	-	(*26*, *27*, *75*)	(*35*, *36*, *38*, *75*)
IODP U1336	128°15.253′W	7°42.067′N	East Pacific	4286	(*39*)	-	(*26*, *27*)	(*36*, *39*)
IODP U1335	126°17.002′W	5°18.735′N	East Pacific	4327.5	(*36*, *39*, *75*)	-	(*26*, *27*, *75*)	(*36*, *39*, *75*)
IODP U1334	131°58.408′W	7°59.998′N	East Pacific	4794	(*40*)	-	(*21*)	(*21*, *37*)
ODP 1218	135°22.00′W	8°53.378′N	East Pacific	4828	(*75*)	-	(*75*)	(*75*)
ODP 851	110°34.3′W	2°46.2′N	East Pacific	3760	(*75*)	-	(*75*)	(*75*)
ODP 850	110°31.3′W	1°17.8′N	East Pacific	3786	(*75*)	-	(*75*)	(*75*)
ODP 849	110°31′W	0°11′N	East Pacific	3851	(*75*)	-	(*75*)	(*75*)
ODP 848	110°28.2′W	1°52.1′S	East Pacific	3868	-	(*72*, *77*)	-	(*72*, *77*)
**DSDP 607	32°57′W	41°00′N	North Atlantic	3427	-	(*69*)	-	(*69*)
ODP 1267	1°43′E	28°6′S	South Atlantic	4355	(*24*, *41*)	-	(*22*, *24*)	(*22*, *24*)
ODP 1264	2°50.730′E	28°31.955′S	South Atlantic	2505	(*9*), *This study*	*This study*	-	(*9*),*This study*

**The full name of DSDP is Deep Sea Drilling Program.

Table S2. Parameters used in the physical scheme

Symbols	Description	Flux (*Sv*)	Symbols	Description	Value
Q_1	Water flow in boxes "N", "D", and "E"	5	Vol_S	Volume of the box "S"	7.5×10^6 km^3
Q_2	Water flow in boxes "E", "I", and "B"	10	Vol_E	Volume of the box "E"	5.4×10^7 km^3
Q_3	Water flow in boxes "S", "I", "B", "D", and "E"	8	Vol_N	Volume of the box "N"	7.5×10^6 km^3
f_{si}	Water mixing between boxes "S" and "I"	80	Vol_I	Volume of the box "I"	8.6×10^7 km^3
f_{ib}	Water mixing between boxes "I" and "B"	60	Vol_D	Volume of the box "D"	7×10^8 km^3
f_{ed}	Water mixing between boxes "E" and "D"	12.5	Vol_B	Volume of the box "B"	4.5×10^8 km^3
f_{db}	Water mixing between boxes "D" and "B"	10	$Area_S$	Area of the surface box "S"	3.7×10^6 km^2
f_{nd}	Water mixing between boxes "N" and "D"	22	$Area_E$	Area of the surface box "E"	2.7×10^8 km^2
f_{se}	Water mixing between boxes "S" and "E"	20	$Area_N$	Area of the surface box "N"	3.7×10^6 km^2
f_{en}	Water mixing between boxes "E" and "N"	20			
f_{id}	Water mixing between boxes "I" and "D"	6.7			

Table S3. Parameters used in the biogeochemical scheme

Parameter	Description	Value	Reference
$r_{Corg:p}$	Redfield ratio of POC	106	(*108*)
$r_{C:P}$	Redfield ratio of total carbon (POC and PIC)	$r_{Corg:p}$/(1-*rain ratio*)	(*108*)
$r_{O_2:P}$	Redfield ratio of oxygen	177	(*108*)
$r_{N:P}$	Redfield ratio of nitrogen	16	(*108*)
$r_{ALK:P}$	Redfield ratio of ALK	2×*rain ratio*×$r_{C:P}$-0.7×$r_{N:P}$	(*108*)
rain ratio	ratio of PIC to POC exported from the surface ocean	0.1	(*12*)
PP_S	Primary productivity in "S"	0.48×10^{12} mol P yr^{-1} m^{-3}	(*12*)
PP_N	Primary productivity in "N"	0.3×10^{12} mol P yr^{-1} m^{-3}	(*12*)
g	Fraction of organic matter that demineralizes in boxes "I" and "D"	0.5	(*102*)
rom	Fraction of organic matter that is buried in sediments	0.01	(*12*)
f_{sil}	Baseline for the weathering of silicates	5×10^{12} mol yr^{-1}	(*12*)
f_{carb}	Baseline for the weathering of carbonates	10.7×10^{12} mol yr^{-1}	(*12*)
$CO_{2,ref}$	Reference value of weathering	400 ppm	(*12*)
α_s	Exponential constant of silicate weathering	0.3	(*99*)
$DG_{volkero}$	Degassing flux from volcanoes and kerogen	7.78×10^{12} mol yr^{-1}	(*12*)
Pv	Piston velocity for ocean-atmosphere exchange	3 m d^{-1}	(*102*)
WDAMP	Damping rate for burial of carbonates in deep water	3×10^{-5} yr^{-1}	(*12*)
CDTARG	Target concentration of carbonate ion in bottom water	85 μmol kg^{-1}	(*102*)
ε_P	Isotopic fractionation of organic carbon	-23‰	(*12*)
$\delta^{13}C_{river}$	Carbon isotope composition of river water	-5‰	(*12*)
$\delta^{13}C_{volkero}$	Carbon isotope composition of volcano and kerogen degassing	-5‰	(*109*)
$rivPO_4$	Riverine input of phosphate	*Wsil*/200	(*12*)

Table S4. Equilibrium values after model spin-up

Variable	Description	Value
$\delta^{13}C_S$	$\delta^{13}C$ of southern surface box "S"	2.55‰
$\delta^{13}C_E$	$\delta^{13}C$ of equatorial box "E"	3.02‰
$\delta^{13}C_N$	$\delta^{13}C$ of northern surface box "N"	2.74‰
$\delta^{13}C_I$	$\delta^{13}C$ of southern intermediate box "I"	2.06‰
$\delta^{13}C_D$	$\delta^{13}C$ of deep box "D"	1.83‰
$\delta^{13}C_B$	$\delta^{13}C$ of bottom box "B"	1.58‰
$[CO_3^{2-}]_S$	Carbonate ion concentration of southern box "S"	166.2 mmol m^{-3}
$[CO_3^{2-}]_E$	Carbonate ion concentration of equatorial box "E"	213.3 mmol m^{-3}
$[CO_3^{2-}]_N$	Carbonate ion concentration of northern box "N"	181.2 mmol m^{-3}
$[CO_3^{2-}]_I$	Carbonate ion concentration of southern intermediate box "I"	136.6 mmol m^{-3}
$[CO_3^{2-}]_D$	Carbonate ion concentration of deep box "D"	125.9 mmol m^{-3}
$[CO_3^{2-}]_B$	Carbonate ion concentration of bottom box "B"	106.3 mmol m^{-3}
pCO_2	Concentration of atmospheric CO_2	399 ppmv
$Carb_{bottom}$	Burial of bottom water carbonates	6.8×10^{12} mol yr^{-1}
POC_{bury}	Burial of organic carbon	2.64×10^{10} mol yr^{-1}

Data S1. (separate file)

Astronomically tuned age model of IODP Site U1505.

Data S2. (separate file)

Benthic foraminiferal $\delta^{18}O$ and $\delta^{13}C$ records of IODP Site U1505.

Data S3. (separate file)

Deep-sea carbonate ion saturation ($\Delta[CO_3^{2-}]$) and phosphate concentration ($[PO_4^{3-}]$) records from IODP Site U1505 over the intervals 24.3-20.9 Ma and 17.4-13.5 Ma.

Data S4. (separate file)

Integrated model simulation results shown in Fig. 4.

Code S1. (separate file)

The code for the seven-box biogeochemical model and the original outputs from all experiments.

REFERENCES

1. L. A. Hinnov, Cyclostratigraphy and its revolutionizing applications in the earth and planetary sciences. *Geol. Soc. Am. Bull.* **125**, 1703–1734 (2013).

2. T. Westerhold, N. Marwan, A. J. Drury, D. Liebrand, C. Agnini, E. Anagnostou, J. S. K. Barnet, S. M. Bohaty, D. De Vleeschouwer, F. Florindo, T. Frederichs, D. A. Hodell, A. E. Holbourn, D. Kroon, V. Lauretano, K. Littler, L. J. Lourens, M. Lyle, H. Pälike, U. Röhl, J. Tian, R. H. Wilkens, P. A. Wilson, J. C. Zachos, An astronomically dated record of Earth's climate and its predictability over the last 66 million years. *Science* **369**, 1383–1387 (2020).

3. J. Zachos, M. Pagani, L. Sloan, E. Thomas, K. Billups, Trends, rhythms, and aberrations in global climate 65 Ma to present. *Science* **292**, 686–693 (2001).

4. B. S. Cramer, J. R. Toggweiler, J. D. Wright, M. E. Katz, K. G. Miller, Ocean overturning since the Late Cretaceous: Inferences from a new benthic foraminiferal isotope compilation. *Paleoceanogr. Paleoclimatol.* **24**, doi.org/10.1029/2008PA001683 (2009).

5. H. Pälike, R. D. Norris, J. O. Herrle, P. A. Wilson, H. K. Coxall, C. H. Lear, N. J. Shackleton, A. K. Tripati, B. S. Wade, The heartbeat of the Oligocene Climate System. *Science* **314**, 1894–1898 (2006).

6. J. Laskar, P. Robutel, F. Joutel, M. Gastineau, A. C. M. Correia, B. Levrard, A long-term numerical solution for the insolation quantities of the Earth. *Astron. Astrophys.* **428**, 261–285 (2004).

7. D. De Vleeschouwer, A. J. Drury, M. Vahlenkamp, F. Rochholz, D. Liebrand, H. Pälike, High-latitude biomes and rock weathering mediate climate–carbon cycle feedbacks on eccentricity timescales. *Nat. Commun.* **11**, 5013 (2020).

8. I. J. Kocken, M. J. Cramwinckel, R. E. Zeebe, J. J. Middelburg, A. Sluijs, The 405 kyr and 2.4 Myr eccentricity components in Cenozoic carbon isotope records. *Clim. Past* **15**, 91–104 (2019).

9. F. Liu, J. Du, E. Huang, W. Ma, X. Ma, L. J. Lourens, J. Tian, Accelerated marine carbon cycling forced by tectonic degassing over the Miocene Climate Optimum. *Sci. Bull.* **69**, 823–832 (2024).

10. S. K. Turner, Pliocene switch in orbital–scale carbon cycle/climate dynamics. *Paleoceanogr. Paleoclimatol.* **29**, 1256–1266 (2014).

11. M. Cao, Z. Wang, Y. Sui, Y. Li, Z. Zhang, A. Xiao, R. Zhang, D. B. Kemp, Mineral dust coupled with climate–carbon cycle on orbital timescales over the past 4 Ma. *Geophys. Res. Lett.* **48**, e2021GL095327 (2021).

12. W. Ma, J. Tian, Q. Li, P. Wang, Simulation of long eccentricity (400-kyr) cycle in ocean carbon reservoir during Miocene Climate Optimum: Weathering and nutrient response to orbital change. *Geophys. Res. Lett.* **38**, doi.org/10.1029/2011GL047680 (2011).

13. X. Ma, W. Ma, J. Tian, J. Yu, E. Huang, Ice sheet and terrestrial input impacts on the 100-kyr ocean carbon cycle during the Middle Miocene. *Glob. Planet. Change* **208**, 103723 (2022).

14. T. Westerhold, U. Röhl, B. Donner, J. C. Zachos, Global extent of Early Eocene hyperthermal events: A new Pacific benthic foraminiferal isotope record from Shatsky Rise (ODP Site 1209). *Paleoceanogr. Paleoclimatol.* **33**, 626–642 (2018).

15. R. E. Zeebe, T. Westerhold, K. Littler, J. C. Zachos, Orbital forcing of the Paleocene and Eocene carbon cycle. *Paleoceanogr. Paleoclimatol.* **32**, 440–465 (2017).

16. P. Wang, Q. Li, J. Tian, Z. Jian, C. Liu, L. Li, W. Ma, Long-term cycles in the carbon reservoir of the Quaternary Ocean: a perspective from the South China Sea. *Natl. Sci. Rev.* **1**, 119–143 (2014).

17. Z. Jian, H.C. Larsen, C.A. Alvarez Zarikian, Z. Sun, J.M. Stock, A. Klaus, J. Boaga, S.A. Bowden, A. Briais, Y. Chen, D. Cukur, K.A. Dadd, W. Ding, M.J. Dorais, E.C. Ferré, F. Ferreira, A. Furusawa, A.J. Gewecke, J.L. Hinojosa, T.W. Höfig, K.-H. Hsiung, B. Huang, E. Huang, X.-L. Huang, S. Jiang, H. Jin, B.G. Johnson, R.M. Kurzawski, C. Lei, B. Li, L. Li, Y. Li, J. Lin, C. Liu, C. Liu, Z. Liu, A. Luna, C. Lupi, A.J. McCarthy, G. Mohn, L.S.

Ningthoujam, M. Nirrengarten, N. Osono, D.W. Peate, P. Persaud, N. Qui, C.M. Robinson, S. Satolli, I. Sauermilch, J.C. Schindlbeck, S.M. Skinner, S.M. Straub, X. Su, L. Tian, F.M. van der Zwan, S. Wan, H. Wu, R. Xiang, R. Yadav, L. Yi, C. Zhang, J. Zhang, Y. Zhang, N. Zhao, G. Zhong, L. Zhong, "Site U1505. South China Sea Rifted Margin", *in Proceedings of the International Ocean Discovery Program 367/368*, Z. Sun, Z. Jian, J.M. Stock, H.C. Larsen, A. Klaus, C.A. Alvarez Zarikian, Expedition 367/368 Scientists, Eds. (International Ocean Discovery Program, College Station, TX, 2018).

18. R. Hall, "The plate tectonics of Cenozoic SE Asia and the distribution of land and sea," in *Biogeography and Geological Evolution of SE Asia*, R. Hall, J. D. Holloway, Eds. (Backhuys Publishers, 1998), pp. 99–131.

19. A. E. Holbourn, W. Kuhnt, S. C. Clemens, K. G. D. Kochhann, J. Jöhnck, J. Lübbers, N. Andersen, Late Miocene climate cooling and intensification of southeast Asian winter monsoon. *Nat. Commun.* **9**, 1584 (2018).

20. P. Wang, W.L. Prell, P. Blum, Leg 184 Shipboard Scientific Party, in *Proceeding of the Ocean Drilling Program 184, Initial Reports* (Ocean Drilling Program, College Station, TX, 2000).

21. H. M. Beddow, D. Liebrand, D. S. Wilson, F. J. Hilgen, A. Sluijs, B. S. Wade, L. J. Lourens, Astronomical tunings of the Oligocene–Miocene transition from Pacific Ocean Site U1334 and implications for the carbon cycle. *Clim. Past* **14**, 255–270 (2018).

22. A. J. Drury, D. Liebrand, T. Westerhold, H. M. Beddow, D. A. Hodell, N. Rohlfs, R. H. Wilkens, M. Lyle, D. B. Bell, D. Kroon, H. Pälike, L. J. Lourens, Climate, cryosphere and carbon cycle controls on Southeast Atlantic orbital-scale carbonate deposition since the Oligocene (30–0 Ma). *Clim. Past* **17**, 2091–2117 (2021).

23. A. Holbourn, W. Kuhnt, D. K. Kulhanek, G. Mountain, Y. Rosenthal, T. Sagawa, J. Lübbers, N. Andersen, Re-organization of Pacific overturning circulation across the Miocene Climate Optimum. *Nat. Commun.* **15**, 8135 (2024).

24. D. Liebrand, H. M. Beddow, L. J. Lourens, H. Pälike, I. Raffi, S. M. Bohaty, F. J. Hilgen, M. J. M. Saes, P. A. Wilson, A. E. van Dijk, D. A. Hodell, D. Kroon, C. E. Huck, S. J. Batenburg,

Cyclostratigraphy and eccentricity tuning of the early Oligocene through early Miocene (30.1–17.1 Ma): *Cibicides mundulus* stable oxygen and carbon isotope records from Walvis Ridge Site 1264. *Earth Planet. Sci. Lett.* **450**, 392–405 (2016).

25. M. Lyle, J. Backman, "Data report: Calibration of XRF-estimated $CaCO_3$ along the Site U1338 splice," in *Proceedings of the Integrated Ocean Drilling Program 320/321*, H. Pälike, M. Lyle, H. Nishi, I. Raffi, K. Gamage, A. Klaus, Expedition 320/321 Scientists, Eds. (Integrated Ocean Drilling Program Management International, Tokyo, 2013).

26. J. K. Shackford, M. Lyle, R. Wilkens, J. Tian, "Data report: Raw and normalized elemental data along the Site U1335, U1336, and U1337 splices from x-ray fluorescence scanning," in *Proceedings of the Integrated Ocean Drilling Program 320/321*, H. Pälike, M. Lyle, H. Nishi, I. Raffi, K. Gamage, A. Klaus, Expedition 320/321 Scientists, Eds. (Integrated Ocean Drilling Program Management International, Tokyo, 2014).

27. J. K. Wilson, "Early Miocene carbonate dissolution in the eastern equatorial Pacific," thesis, Texas A&M University (2014).

28. Z. Jian, H. Jin, M. A. Kaminski, F. Ferreira, B. Li, P.-S. Yu, Discovery of the marine Eocene in the northern South China Sea. *Natl. Sci. Rev.* **6**, 881–885 (2019).

29. J. Yu, H. Elderfield, Benthic foraminiferal B/Ca ratios reflect deep water carbonate saturation state. *Earth Planet. Sci. Lett.* **258**, 73–86 (2007).

30. E. A. Boyle, Cadmium and $\delta^{13}C$ paleochemical ocean distributions during the stage 2 glacial maximum. *Annu. Rev. Earth Planet. Sci.* **20**, 245–287 (1992).

31. T. D. Herbert, C. A. Dalton, Z. Liu, A. Salazar, W. Si, D. S. Wilson, Tectonic degassing drove global temperature trends since 20 Ma. *Science* **377**, 116–119 (2022).

32. J. Kasbohm, B. Schoene, Rapid eruption of the Columbia River flood basalt and correlation with the mid-Miocene climate optimum. *Sci. Adv.* **4**, eaat8223 (2018).

33. X. Tian, W. R. Buck, Intrusions induce global warming before continental flood basalt volcanism. *Nat. Geosci.* **15**, 417–422 (2022).

34. A. Holbourn, W. Kuhnt, M. Lyle, L. Schneider, O. Romero, N. Andersen, Middle Miocene climate cooling linked to intensification of eastern equatorial Pacific upwelling. *Geology* **42**, 19–22 (2014).

35. A. Holbourn, W. Kuhnt, K. G. D. Kochhann, N. Andersen, K. J. Sebastian Meier, Global perturbation of the carbon cycle at the onset of the Miocene Climatic Optimum. *Geology* **43**, 123–126 (2015).

36. K. G. D. Kochhann, A. Holbourn, W. Kuhnt, J. E. T. Channell, M. Lyle, J. K. Shackford, R. H. Wilkens, N. Andersen, Eccentricity pacing of eastern equatorial Pacific carbonate dissolution cycles during the Miocene Climatic Optimum. *Paleoceanogr. Paleoclimatol.* **31**, 1176–1192 (2016).

37. D. Liebrand, B. S. Wade, H. M. Beddow, D. J. King, A. D. Harrison, H. J. H. Johnstone, A. J. Drury, H. Pälike, A. Sluijs, L. J. Lourens, Oceanography of the Eastern Equatorial Pacific Ocean Across the Oligocene–Miocene Transition. *Paleoceanogr. Paleoclimatol.* **39**, e2024PA004892 (2024).

38. J. Tian, M. Yang, M. W. Lyle, R. Wilkens, J. K. Shackford, Obliquity and long eccentricity pacing of the Middle Miocene climate transition. *Geochem. Geophys. Geosyst.* **14**, 1740–1755 (2013).

39. J. Voigt, E. C. Hathorne, M. Frank, A. Holbourn, Minimal influence of recrystallization on middle Miocene benthic foraminiferal stable isotope stratigraphy in the eastern equatorial Pacific. *Paleoceanogr. Paleoclimatol.* **31**, 98–114 (2016).

40. H. M. Beddow, D. Liebrand, A. Sluijs, B. S. Wade, L. J. Lourens, Global change across the Oligocene–Miocene transition: High-resolution stable isotope records from IODP Site U1334 (equatorial Pacific Ocean). *Paleoceanogr. Paleoclimatol.* **31**, 81–97 (2016).

41. D. Liebrand, L. J. Lourens, D. A. Hodell, B. de Boer, R. S. W. van de Wal, H. Pälike, Antarctic ice sheet and oceanographic response to eccentricity forcing during the early Miocene. *Clim. Past* **7**, 869–880 (2011).

42. M. Raitzsch, J. Bijma, T. Bickert, M. Schulz, A. Holbourn, M. Kučera, Atmospheric carbon dioxide variations across the middle Miocene climate transition. *Clim. Past* **17**, 703–719 (2021).

43. A. Holbourn, W. Kuhnt, S. C. Clemens, D. Heslop, A □12 Myr Miocene Record of East Asian monsoon variability from the South China Sea. *Paleoceanogr. Paleoclimatol.* **36**, e2021PA004267 (2021).

44. C.-S. Jin, D. Xu, M. Li, P. Hu, Z. Jiang, J. Liu, Y. Miao, F. Wu, W. Liang, Q. Zhang, B. Su, Q. Liu, R. Zhang, J. Sun, Tectonic and orbital forcing of the South Asian monsoon in central Tibet during the late Oligocene. *Proc. Natl. Acad. Sci. U.S.A.* **120**, e2214558120 (2023).

45. F. T. Mackenzie, J. W. Morse, Sedimentary carbonates through Phanerozoic time. *Geochim. Cosmochim. Acta* **56**, 3281–3295 (1992).

46. J. D. Milliman, A. W. Droxler, Neritic and pelagic carbonate sedimentation in the marine environment: ignorance is not bliss. *Geol. Rundsch.* **85**, 496–504 (1996).

47. K. G. Miller, J. V. Browning, W. J. Schmelz, R. E. Kopp, G. S. Mountain, J. D. Wright, Cenozoic sea-level and cryospheric evolution from deep-sea geochemical and continental margin records. *Sci. Adv.* **6**, eaaz1346 (2020).

48. A. Holbourn, W. Kuhnt, K. G. D. Kochhann, K. M. Matsuzaki, N. Andersen, "Middle Miocene climate–carbon cycle dynamics: Keys for understanding future trends on a warmer Earth?," in *Understanding the Monterey Formation and Similar Biosiliceous Units across Space and Time*, I. W. Aiello, J. A. Barron, A. C. Ravelo, Eds. (Geological Society of America, 2022), pp. 93–111.

49. R. E. Zeebe, D. Wolf-Gladrow, *CO_2 in Seawater: Equilibrium, Kinetics, Isotopes* (Gulf Professional Publishing, 2001).

50. L. R. Kump, Interpreting carbon-isotope excursions: Strangelove oceans. *Geology* **19**, 299–302 (1991).

51. M. Liu, P. A. Raymond, R. Lauerwald, Q. Zhang, G. Trapp-Müller, K. L. Davis, N. Moosdorf, C. Xiao, J. J. Middelburg, A. F. Bouwman, Global riverine land-to-ocean carbon export constrained by observations and multi-model assessment. *Nat. Geosci.* **17**, 896–904 (2024).

52. T. R. Marwick, F. Tamooh, C. R. Teodoru, A. V. Borges, F. Darchambeau, S. Bouillon, The age of river-transported carbon: A global perspective. *Glob. Biogeochem. Cycles* **29**, 122–137 (2015).

53. S. Shan, C. Luo, Y. Qi, W.-J. Cai, S. Sun, D. Fan, X. Wang, Carbon isotopic and lithologic constraints on the sources and cycling of inorganic carbon in four large rivers in China: Yangtze, Yellow, Pearl, and Heilongjiang. *J. Geophys. Res. Biogeosci.* **126**, e2020JG005901 (2021).

54. A. K. Hilting, L. R. Kump, T. J. Bralower, Variations in the oceanic vertical carbon isotope gradient and their implications for the Paleocene-Eocene biological pump. *Paleoceanography* **23**, PA3222 (2008).

55. W. H. Berger, Increase of carbon dioxide in the atmosphere during deglaciation: The coral reef hypothesis. *Naturwissenschaften* **69**, 87–88 (1982).

56. W. H. Berger, Deglacial CO_2 buildup: Constraints on the coral-reef model. *Palaeogeogr. Palaeoclimatol. Palaeoecol.* **40**, 235–253 (1982).

57. B. N. Opdyke, J. C. Walker, Return of the coral reef hypothesis: Basin to shelf partitioning of $CaCO_3$ and its effect on atmospheric CO_2. *Geology* **20**, 733–736 (1992).

58. K. Littler, U. Röhl, T. Westerhold, J. C. Zachos, A high-resolution benthic stable-isotope record for the South Atlantic: Implications for orbital-scale changes in Late Paleocene–Early Eocene climate and carbon cycling. *Earth Planet. Sci. Lett.* **401**, 18–30 (2014).

59. L. J. Lourens, A. Sluijs, D. Kroon, J. C. Zachos, E. Thomas, U. Röhl, J. Bowles, I. Raffi, Astronomical pacing of late Palaeocene to early Eocene global warming events. *Nature* **435**, 1083–1087 (2005).

60. J. C. Zachos, U. Röhl, S. A. Schellenberg, A. Sluijs, D. A. Hodell, D. C. Kelly, E. Thomas, M. Nicolo, I. Raffi, L. J. Lourens, H. McCarren, D. Kroon, Rapid acidification of the ocean during the Paleocene-Eocene thermal maximum. *Science* **308**, 1611–1615 (2005).

61. J. C. Zachos, H. McCarren, B. Murphy, U. Röhl, T. Westerhold, Tempo and scale of late Paleocene and early Eocene carbon isotope cycles: Implications for the origin of hyperthermals. *Earth Planet. Sci. Lett.* **299**, 242–249 (2010).

62. V. Lauretano, K. Littler, M. Polling, J. C. Zachos, L. J. Lourens, Frequency, magnitude and character of hyperthermal events at the onset of the Early Eocene Climatic Optimum. *Clim. Past* **11**, 1313–1324 (2015).

63. M. Gutjahr, A. Ridgwell, P. F. Sexton, E. Anagnostou, P. N. Pearson, H. Pälike, R. D. Norris, E. Thomas, G. L. Foster, Very large release of mostly volcanic carbon during the Palaeocene–Eocene Thermal Maximum. *Nature* **548**, 573–577 (2017).

64. J. C. Zachos, G. R. Dickens, R. E. Zeebe, An early Cenozoic perspective on greenhouse warming and carbon-cycle dynamics. *Nature* **451**, 279–283 (2008).

65. D. Liebrand, A. T. M. de Bakker, H. M. Beddow, P. A. Wilson, S. M. Bohaty, G. Ruessink, H. Pälike, S. J. Batenburg, F. J. Hilgen, D. A. Hodell, C. E. Huck, D. Kroon, I. Raffi, M. J. M. Saes, A. E. van Dijk, L. J. Lourens, Evolution of the early Antarctic ice ages. *Proc. Natl. Acad. Sci. U.S.A.* **114**, 3867–3872 (2017).

66. T. E. van Peer, D. Liebrand, V. E. Taylor, S. Brzelinski, I. Wolf, A. Bornemann, O. Friedrich, S. M. Bohaty, C. Xuan, P. C. Lippert, P. A. Wilson, Eccentricity pacing and rapid termination of the early Antarctic ice ages. *Nat. Commun.* **15**, 10600 (2024).

67. J. Zachos, N. J. Shackleton, J. S. Revenaugh, H. Pälike, B. P. Flower, Climate response to orbital forcing across the Oligocene–Miocene boundary. *Science* **292**, 274–278 (2001).

68. D. B. Bell, S. J. A. Jung, D. Kroon, L. J. Lourens, D. A. Hodell, Local and regional trends in Plio–Pleistocene $\delta^{18}O$ records from benthic foraminifera. *Geochem. Geophys. Geosyst.* **15**, 3304–3321 (2014).

69. J. R. Farmer, B. Hönisch, L. L. Haynes, D. Kroon, S. Jung, H. L. Ford, M. E. Raymo, M. Jaume-Seguí, D. B. Bell, S. L. Goldstein, L. D. Pena, M. Yehudai, J. Kim, Deep Atlantic Ocean carbon storage and the rise of 100,000-year glacial cycles. *Nat. Geosci.* **12**, 355–360 (2019).

70. Z. Jian, H. Dang, J. Yu, Q. Wu, X. Gong, C. Stepanek, C. Colin, L. Hu, G. Lohmann, X. Zhou, S. Wan, Changes in deep Pacific circulation and carbon storage during the Pliocene–Pleistocene transition. *Earth Planet. Sci. Lett.* **605**, 118020 (2023).

71. J. Kerr, R. Rickaby, J. Yu, H. Elderfield, A. Y. Sadekov, The effect of ocean alkalinity and carbon transfer on deep-sea carbonate ion concentration during the past five glacial cycles. *Earth Planet. Sci. Lett.* **471**, 42–53 (2017).

72. C. H. Lear, K. Billups, R. E. M. Rickaby, L. Diester-Haass, E. M. Mawbey, S. M. Sosdian, Breathing more deeply: Deep ocean carbon storage during the mid-Pleistocene climate transition. *Geology* **44**, 1035–1038 (2016).

73. L. E. Lisiecki, M. E. Raymo, A Pliocene–Pleistocene stack of 57 globally distributed benthic $\delta^{18}O$ records. *Paleoceanography* **20**, PA1003 (2005).

74. M. Lyle, A. Olivarez Lyle, T. Gorgas, A. Holbourn, T. Westerhold, E. Hathorne, K. Kimoto, S. Yamamoto, "Data report: Raw and normalized elemental data along the Site U1338 splice from x-ray fluorescence scanning," in *Proceedings of the Integrated Ocean Drilling Program* 320/321, H. Pälike, M. Lyle, H. Nishi, I. Raffi, K. Gamage, A. Klaus, Expedition 320/321 Scientists, Eds. (Integrated Ocean Drilling Program Management International, Tokyo, 2012).

75. M. Lyle, A. J. Drury, J. Tian, R. Wilkens, T. Westerhold, Late Miocene to Holocene high-resolution eastern equatorial Pacific carbonate records: stratigraphy linked by dissolution and paleoproductivity. *Clim. Past* **15**, 1715–1739 (2019).

76. B. Qin, Z. Xiong, T. J. Algeo, Q. Jia, D. Nürnberg, T. Li, Obliquity Pacing of Deep Pacific Carbonate Chemistry During the Plio–Pleistocene. *Geophys. Res. Lett.* **51**, e2024GL110093 (2024).

77. S. M. Sosdian, Y. Rosenthal, J. R. Toggweiler, Deep Atlantic carbonate ion and $CaCO_3$ compensation during the Ice Ages. *Paleoceanogr. Paleoclimatol.* **33**, 546–562 (2018).

78. S. M. White, A. C. Ravelo, The Benthic B/Ca Record at Site 806: New constraints on the temperature of the West Pacific warm pool and the "El Padre" State in the Pliocene. *Paleoceanogr. Paleoclimatol.* **35**, e2019PA003812 (2020).

79. S. Kotov, H. Pälike, QAnalySeries—A cross-platform time series tuning and analysis tool (AGU Fall Meeting Abstracts, abstract PP53D-1230, 2018).

80. D. Paillard, L. Labeyrie, P. Yiou, Macintosh Program performs time-series analysis. *Eos Trans. AGU* **77**, 379 (1996).

81. E. A. Boyle, L. D. Keigwin, Comparison of Atlantic and Pacific paleochemical records for the last 215,000 years: Changes in deep ocean circulation and chemical inventories. *Earth Planet. Sci. Lett.* **76**, 135–150 (1985).

82. P. A. Martin, D. W. Lea, A simple evaluation of cleaning procedures on fossil benthic foraminiferal Mg/Ca. *Geochem. Geophys. Geosyst.* **3**, 8401 (2002).

83. J. W. B. Rae, G. L. Foster, D. N. Schmidt, T. Elliott, Boron isotopes and B/Ca in benthic foraminifera: Proxies for the deep ocean carbonate system. *Earth Planet. Sci. Lett.* **302**, 403–413 (2011).

84. M. Raitzsch, E. C. Hathorne, H. Kuhnert, J. Groeneveld, T. Bickert, Modern and late Pleistocene B/Ca ratios of the benthic foraminifer *Planulina wuellerstorfi* determined with laser ablation ICP-MS. *Geology* **39**, 1039–1042 (2011).

85. J. Yu, R. F. Anderson, Z. Jin, J. W. B. Rae, B. N. Opdyke, S. M. Eggins, Responses of the deep ocean carbonate system to carbon reorganization during the Last Glacial–interglacial cycle. *Quat. Sci. Rev.* **76**, 39–52 (2013).

86. J. Yu, R. Anderson, E. Rohling, Deep ocean carbonate chemistry and glacial–interglacial atmospheric CO_2 change. *Oceanography* **27**, 16–25 (2014).

87. D. Lemarchand, J. Gaillardet, É. Lewin, C. J. Allègre, The influence of rivers on marine boron isotopes and implications for reconstructing past ocean pH. *Nature* **408**, 951–954 (2000).

88. D. Lemarchand, J. Gaillardet, É. Lewin, C. J. Allègre, Boron isotope systematics in large rivers: implications for the marine boron budget and paleo-pH reconstruction over the Cenozoic. *Chem. Geol.* **190**, 123–140 (2002).

89. L. Simon, C. Lécuyer, C. Maréchal, N. Coltice, Modelling the geochemical cycle of boron: Implications for the long-term $\delta^{11}B$ evolution of seawater and oceanic crust. *Chem. Geol.* **225**, 61–76 (2006).

90. A. J. Spivack, M. R. Palmer, J. M. Edmond, The sedimentary cycle of the boron isotopes. *Geochim. Cosmochim. Acta* **51**, 1939–1949 (1987).

91. W. S. Broecker, Glacial to interglacial changes in ocean chemistry. *Prog. Oceanogr.* **11**, 151–197 (1982).

92. L. L. Haynes, B. Hönisch, K. A. Dyez, K. Holland, Y. Rosenthal, C. R. Fish, A. V. Subhas, J. W. B. Rae, Calibration of the B/Ca proxy in the planktic foraminifer *Orbulina universa* to Paleocene seawater conditions. *Paleoceanography* **32**, 580–599 (2017).

93. E. A. Boyle, Cadmium: Chemical tracer of deepwater paleoceanography. *Paleoceanography* **3**, 471–489 (1988).

94. H. J. W. de Baar, P. M. Saager, R. F. Nolting, J. van der Meer, Cadmium versus phosphate in the world ocean. *Mar. Chem.* **46**, 261–281 (1994).

95. H. Elderfield, R. E. M. Rickaby, Oceanic Cd/P ratio and nutrient utilization in the glacial Southern Ocean. *Nature* **405**, 305–310 (2000).

96. The GEOTRACES Group, The GEOTRACES Intermediate Data Product 2014. *Mar. Chem.* **177**, 1–8 (2015).

97. R. Schlitzer, R. F. Anderson, E. M. Dodas, M. Lohan, W. Geibert, A. Tagliabue, A. Bowie, C. Jeandel, M. T. Maldonado, W. M. Landing, D. Cockwell, C. Abadie, W. Abouchami, E. P. Achterberg, A. Agather, A. Aguliar-Islas, H. M. van Aken, M. Andersen, C. Archer, M. Auro, H. J. de Baar, O. Baars, A. R. Baker, K. Bakker, C. Basak, M. Baskaran, N. R. Bates, D. Bauch, P. van Beek, M. K. Behrens, E. Black, K. Bluhm, L. Bopp, H. Bouman, K. Bowman, J. Bown, P. Boyd, M. Boye, E. A. Boyle, P. Branellec, L. Bridgestock, G. Brissebrat, T. Browning, K. W. Bruland, H.-J. Brumsack, M. Brzezinski, C. S. Buck, K. N. Buck, K. Buesseler, A. Bull, E. Butler, P. Cai, P. C. Mor, D. Cardinal, C. Carlson, G. Carrasco, N. Casacuberta, K. L. Casciotti, M. Castrillejo, E. Chamizo, R. Chance, M. A. Charette, J. E. Chaves, H. Cheng, F. Chever, M. Christl, T. M. Church, I. Closset, A. Colman, T. M. Conway, D. Cossa, P. Croot, J. T. Cullen, G. A. Cutter, C. Daniels, F. Dehairs, F. Deng, H. T. Dieu, B. Duggan, G. Dulaquais, C. Dumousseaud, Y. Echegoyen-Sanz, R. L. Edwards, M. Ellwood, E. Fahrbach, J. N. Fitzsimmons, A. R. Flegal, M. Q. Fleisher, T. van de Flierdt, M. Frank, J. Friedrich, F. Fripiat, H. Fröllje, S. J. G. Galer, T. Gamo, R. S. Ganeshram, J. Garcia-Orellana, E. Garcia-Solsona, M. Gault-Ringold, E. George, L. J. A. Gerringa, M. Gilbert, J. M. Godoy, S. L. Goldstein, S. R. Gonzalez, K. Grissom, C. Hammerschmidt, A. Hartman, C. S. Hassler, E. C. Hathorne, M. Hatta, N. Hawco, C. T. Hayes, L.-E. Heimbürger, J. Helgoe, M. Heller, G. M. Henderson, P. B. Henderson, S. van Heuven, P. Ho, T. J. Horner, Y.-T. Hsieh, K.-F. Huang, M. P. Humphreys, K. Isshiki, J. E. Jacquot, D. J. Janssen, W. J. Jenkins, S. John, E. M. Jones, J. L. Jones, D. C. Kadko, R. Kayser, T. C. Kenna, R. Khondoker, T. Kim, L. Kipp, J. K. Klar, M. Klunder, S. Kretschmer, Y. Kumamoto, P. Laan, M. Labatut, F. Lacan, P. J. Lam, M. Lambelet, C. H. Lamborg, F. A. C. Le Moigne, E. Le Roy, O. J. Lechtenfeld, J.-M. Lee, P. Lherminier, S. Little, M. López-Lora, Y. Lu, P. Masque, E. Mawji, C. R. Mcclain, C. Measures, S. Mehic, J.-L. M. Barraqueta, P. van der Merwe, R. Middag, S. Mieruch, A. Milne, T. Minami, J. W. Moffett, G. Moncoiffe, W. S. Moore, P. J. Morris, P. L. Morton, Y. Nakaguchi, N. Nakayama, J. Niedermiller, J. Nishioka, A. Nishiuchi, A. Noble, H. Obata, S. Ober, D. C. Ohnemus, J. van Ooijen, J. O'Sullivan, S. Owens, K. Pahnke, M. Paul, F. Pavia, L. D. Pena, B. Peters, F. Planchon, H. Planquette, C. Pradoux, V. Puigcorbé, P. Quay, F. Queroue, A. Radic, S. Rauschenberg, M. Rehkämper, R. Rember, T. Remenyi, J. A. Resing, J. Rickli, S. Rigaud, M. J. A. Rijkenberg, S. Rintoul, L. F. Robinson, M. Roca-Martí, V. Rodellas, T. Roeske, J. M. Rolison, M. Rosenberg, S. Roshan, M. M. R. van der Loeff, E. Ryabenko, M. A. Saito, L. A. Salt, V. Sanial, G. Sarthou, C. Schallenberg, U. Schauer, H.

Scher, C. Schlosser, B. Schnetger, P. Scott, P. N. Sedwick, I. Semiletov, R. Shelley, R. M. Sherrell, A. M. Shiller, D. M. Sigman, S. K. Singh, H. A. Slagter, E. Slater, W. M. Smethie, H. Snaith, Y. Sohrin, B. Sohst, J. E. Sonke, S. Speich, R. Steinfeldt, G. Stewart, T. Stichel, C. H. Stirling, J. Stutsman, G. J. Swarr, J. H. Swift, A. Thomas, K. Thorne, C. P. Till, R. Till, A. T. Townsend, E. Townsend, R. Tuerena, B. S. Twining, D. Vance, S. Velazquez, C. Venchiarutti, M. Villa-Alfageme, S. M. Vivancos, A. H. L. Voelker, B. Wake, M. J. Warner, R. Watson, E. van Weerlee, M. A. Weigand, Y. Weinstein, D. Weiss, A. Wisotzki, E. M. S. Woodward, J. Wu, Y. Wu, K. Wuttig, N. Wyatt, Y. Xiang, R. C. Xie, Z. Xue, H. Yoshikawa, J. Zhang, P. Zhang, Y. Zhao, L. Zheng, X.-Y. Zheng, M. Zieringer, L. A. Zimmer, P. Ziveri, P. Zunino, C. Zurbrick, The GEOTRACES Intermediate Data Product 2017. *Chem. Geol.* **493**, 210–223 (2018).

98. M. Li, L. Hinnov, L. Kump, Acycle: Time-series analysis software for paleoclimate research and education. *Comput. Geosci.* **127**, 12–22 (2019).

99. J. C. Walker, J. F. Kasting, Effects of fuel and forest conservation on future levels of atmospheric carbon dioxide. *Glob. Planet. Change* **5**, 151–189 (1992).

100. R. A. Berner, *The Phanerozoic Carbon Cycle: CO_2 and O_2* (Oxford Univ. Press, 2004); 10.1093/oso/9780195173338.001.0001.

101. J. R. Toggweiler, Variation of atmospheric CO_2 by ventilation of the ocean's deepest water. *Paleoceanography* **14**, 571–588 (1999).

102. J. R. Toggweiler, Origin of the 100,000-year timescale in Antarctic temperatures and atmospheric CO_2. *Paleoceanogr. Paleoclimatol.* **23**, doi.org/10.1029/2006PA001405 (2008).

103. T. Russon, D. Paillard, M. Elliot, Potential origins of 400–500 kyr periodicities in the ocean carbon cycle: A box model approach. *Glob. Biogeochem. Cycles* **24**, doi.org/10.1029/2009GB003586 (2010).

104. Y. Yamanaka, E. Tajika, The role of the vertical fluxes of particulate organic matter and calcite in the oceanic carbon cycle: Studies using an ocean biogeochemical general circulation model. *Glob. Biogeochem. Cycles* **10**, 361–382 (1996).

105. T. E. Cerling, J. M. Harris, B. J. MacFadden, M. G. Leakey, J. Quade, V. Eisenmann, J. R. Ehleringer, Global vegetation change through the Miocene/Pliocene boundary. *Nature* **389**, 153–158 (1997).

106. M. J. Kohn, Carbon isotope compositions of terrestrial C_3 plants as indicators of (paleo) ecology and (paleo)climate. *Proc. Natl. Acad. Sci. U.S.A.* **107**, 19691–19695 (2010).

107. M. J. Whiticar, Stable isotope geochemistry of coals, humic kerogens and related natural gases. *Int. J. Coal Geol.* **32**, 191–215 (1996).

108. A. J. Ridgwell, "Glacial–interglacial perturbations in the global carbon cycle," thesis, University of East Anglia (2001).

109. L. R. Kump, M. A. Arthur, Interpreting carbon-isotope excursions: carbonates and organic matter. *Chem. Geol.* **161**, 181–198 (1999).
